# Large mammal declines and the incipient loss of mammal-bird mutualisms in an African savanna ecosystem

**DOI:** 10.1371/journal.pone.0202536

**Published:** 2018-08-28

**Authors:** Nathan Diplock, Kate Johnston, Antoine Mellon, Laura Mitchell, Madison Moore, Daniel Schneider, Alyssa Taylor, Jess Whitney, Kera Zegar, John Kioko, Christian Kiffner

**Affiliations:** 1 Center for Wildlife Management Studies, The School for Field Studies, Karatu, Tanzania; 2 Department of Biology, Augsburg University, Minneapolis, Minnesota, United States of America; 3 Department of Biological Sciences, Southern Methodist University, Dallas, Texas, United States of America; 4 Department of Biology, University of Vermont, Burlington, Vermont, United States of America; 5 Department of Biological Foundations of Behavior, Franklin and Marshall College, Lancaster, Pennsylvania, United States of America; 6 Department of Environmental Science, Muhlenberg College, Allentown, Pennsylvania, United States of America; 7 Department of Biology, Elizabethtown College, Elizabethtown, Pennsylvania, United States of America; 8 Department of Environmental Conservation, University of Massachusetts, Amherst, Massachusetts, United States of America; 9 College of the Environment, University of Washington, Seattle, Washington, United States of America; Wageningen Universiteit, NETHERLANDS

## Abstract

Over the past half-century, large mammal populations have declined substantially throughout East Africa, mainly due to habitat loss and unsustainable direct exploitation. While it has been acknowledged that the loss of large mammals can have direct and cascading effects on community composition and ecosystem characteristics, limited quantitative work has been done on how declines of large herbivore populations impacts the abundance of mutualistic symbionts. Using a space-for-time observational approach, we quantified the large mammal community alongside the densities, host preferences and behaviors of mutualistic red-billed oxpeckers (*Buphagus erythrorhynchus*), and yellow-billed oxpeckers (*Buphagus africanus*) in northern Tanzania. At the landscape scale, mammal community composition was substantially less diverse in highly human-dominated areas when compared with more protected areas, with an observed complete loss of large wild mammal species in two study areas. Mirroring this trend, oxpecker densities were lowest in the least protected areas, and highest in fully protected areas. Using resource selection functions implemented via generalized linear models at different scales, we found that oxpeckers (1) were predominantly (67% of red-billed oxpeckers; 70% of yellow-billed oxpeckers) feeding on larger (between 500kg and 1500kg) ungulate host species within the mammal community, (2) usually preferred feeding on larger individuals (adults and males) within a specific host species population, and (3) preferred hosts that were more tolerant of their presence. In particular, cattle were especially intolerant of oxpecker presence and were relatively effective in displacing oxpeckers. We found little evidence that oxpecker feeding was parasitic across all host species; wound feeding was only observed on giraffe, comprising 6% and 4% of feeding behavior in red-billed and yellow-billed oxpeckers respectively. Thus, a loss of large-bodied and oxpecker tolerant host species is a likely explanation for declines of oxpecker populations in human dominated landscapes, which may have further cascading effects.

## Introduction

The decline and/or loss of large mammals can have cascading effects on ecosystems, which can prompt ecological, economic, and socio-cultural consequences [1–3]. Long-term data suggest that large mammal populations in East Africa have been reduced by more than 50% over the past half-century [4]. Whereas wildlife declines have substantial and obvious economic and socio-cultural effects such as lower animal protein supply to local populations [5] and the decline of regional tourism [6], indirect ecological effects can be difficult to predict due to complex interspecific interactions and often-unexpected feedback loops between animals and their environment [1, 2, 7]. For example, losses of large mammal populations can have cascading effects on vegetation structure within a habitat through direct utilization [8] and seed dispersal [9, 10], although the direction and strength of these interactions can be variable [1].

Large terrestrial herbivore-vegetation feedback loops have provoked substantial scientific curiosity, but surprisingly little is known on how large mammal declines affect mutualistic species that have coevolved with large mammal hosts. Paleontological research has revealed several avian coextinctions associated with the loss of commensalistic megafauna during the Pleistocene/Holocene transition [1, 11]. However, examples of contemporary coextinctions are sparse, and coextinction predictions are often made utilizing incomplete information about an associated species’ life history and host specificity [12].

It has been conjectured that two of best predictors of a mutualistic species’ coextinction vulnerability are the strength of its host preference, and the potential for plasticity outside of preferred hosts that will allow the species to maintain adequate fitness [13]. Red-billed and yellow-billed oxpeckers (*Buphagus erythrorhynchus*; RBO, *Buphagus africanus*; YBO) provide one of the most conspicuous contemporary examples of mutualistic bird mammal associations [14, 15]. Both RBO and YBO have a demonstrated dependence on large terrestrial herbivore hosts due to their highly specialized life history and narrow host preference [16–18]. It has been demonstrated that oxpeckers predominantly forage on ectoparasites found on large mammalian host species [15, 17, 19, 20], although there is some evidence that RBO and YBO feeding may prolong wound healing, in addition to removing blood and other tissue from associated hosts [21]. The highly specific oxpecker preferences for large mammal hosts is likely the result of high ectoparasite abundance on large mammals which is typically positively scaled with host body mass [22–26], and a host tolerance for RBO and YBO feeding [27–29]. Thus, large mammal declines may have severe cascading effects on oxpecker populations [13].

Throughout sub-Saharan Africa RBO and YBO declines and local extinctions have been documented, particularly outside of protected areas, where large wild mammal populations have widely been replaced with livestock herds [17, 18, 30, 31]. In Southern Africa, it has been conjectured that substituting livestock for wildlife on a landscape scale has caused RBO and YBO declines [18, 30]. To assess the impact of landscape scale abundance of livestock and wildlife on oxpecker density in an East African savanna, we used a space-for-time observational approach across areas ranging from low to high conservation status in northern Tanzania [32]. Subsequently, we used resource selection functions [33] to assess RBO feeding preferences at the mammal community, host population, and individual host scale. This approach of linking probability of use can provide an explanation for variation in RBO and YBO density in the landscape, and evaluate the potential for host plasticity [34, 35]. We documented visible wound presence or absence for all host individuals in addition to feeding behavior to evaluate potential parasitic exploitation by RBOs and YBOs [19, 21]. Finally, we investigated if host species behavior towards oxpeckers can explain host preferences and landscape scale distribution of oxpeckers.

Based on studies of oxpecker distribution in Southern Africa [18, 30] and mammal distribution within our study area in northern Tanzania [32, 36], we expect RBO and YBO density to be lowest in the least-protected study area, and to be positively correlated with the density of preferred hosts within the landscape. At the mammal community level, we anticipate that RBO and YBO feeding preferences are positively associated with host species’ body mass [15, 16, 27–29] up to a body mass threshold [37]. This prediction is based on optimal foraging theory [38, 39]; previous research indicates that ectoparasite abundance (i.e. food availability for oxpeckers) is positively scaled with host body mass [22–26], and that large mammals demonstrate a high tolerance for RBO feeding [27–29, 40]. At the population level, we hypothesized that group size would either reduce oxpecker presence per host via the individual dilution effect [41, 42] or increase oxpecker presence if larger host groups were generally more attractive to oxpeckers. At the individual scale, again following optimal foraging theory, we hypothesized that RBOs and YBOs would prefer larger individuals (i.e. adults over juveniles) and prefer host individuals of the heavier sex in species with substantial sexual size dimorphism [43]. Finally, we expect highly preferred host species to show a high tolerance for oxpecker presence, and non-preferred species to exhibit low tolerance towards oxpeckers.

## Material and methods

This observational study was carried out with approval from TAWIRI, and COSTECH (permits 2016-349-NA-2013-191 and 2017-288-ER-2013-191).

### Study area

We conducted our study in the fragmented Tarangire-Manyara Ecosystem (TME) of Northern Tanzania. The vegetation throughout the TME consists primarily of grassland and open Acacia-Commiphora savanna, although agriculture is widespread in human dominated areas [44]. The climate of the TME is defined as semi-arid; annual precipitation ranges from 415 to 995 mm and mainly occurs during the long rainy season (February-May) and the short rains (November-December) [45, 46].

**Fig 1:**
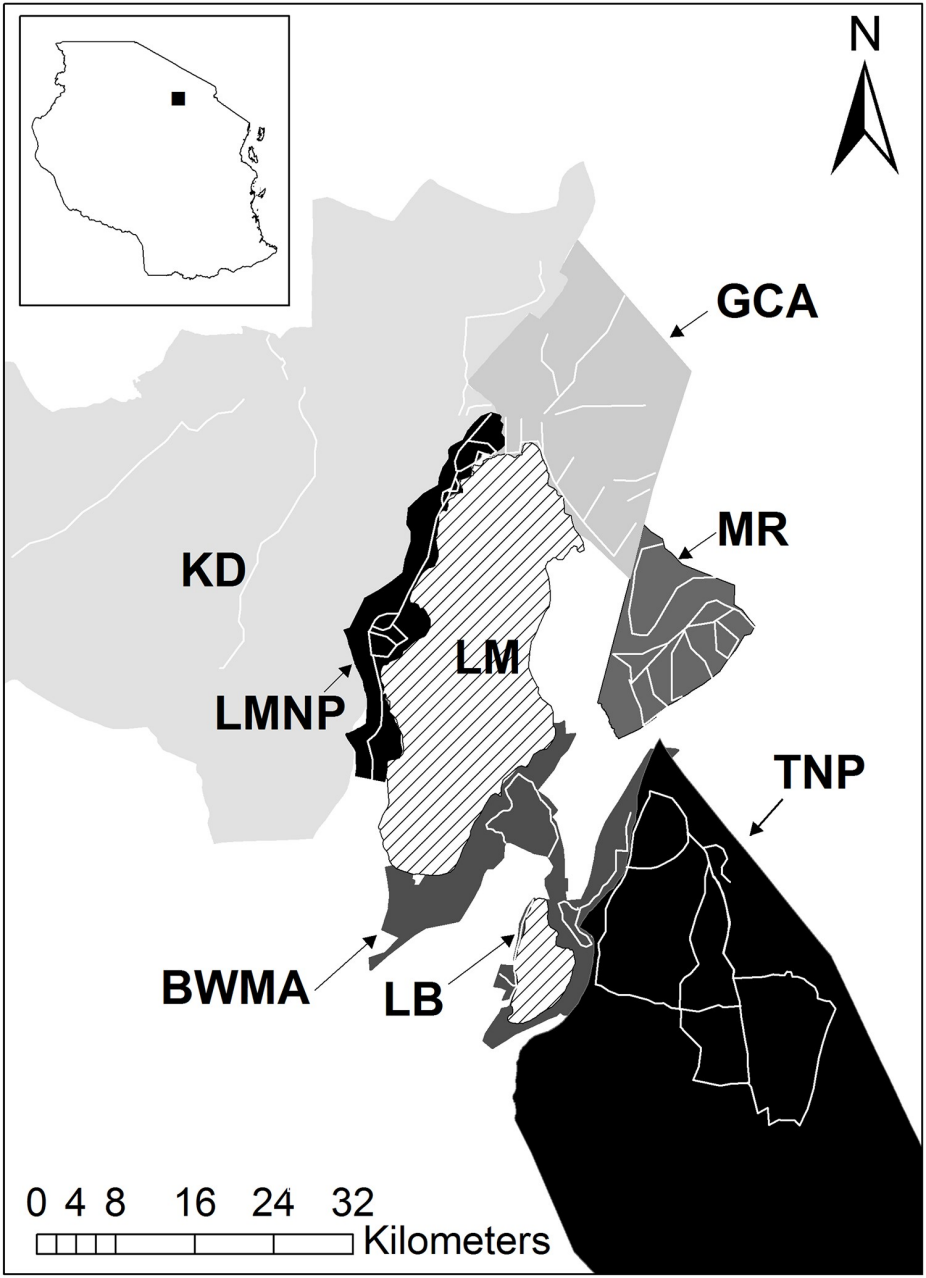
Study areas in northern Tanzania. Oxpecker densities and host preferences were investigated along road transects (white lines) in Tarangire National Park (TNP), Lake Manyara National Park (LMNP), Manyara Ranch (MR), Burunge Wildlife Management Area (BWMA), Mto wa Mbu Game Controlled Area (GCA), and Karatu District (KD). For orientation, we included the location of Lake Manyara (LM) and Lake Burunge (LB). The inset in the top left shows the location of the study area within Tanzania.

This study was conducted in six distinct units of the TME: Karatu District (KD), Mto wa Mbu Game Controlled Area (GCA), Manyara Ranch (MR), Burunge Wildlife Management Area (BWMA), Tarangire National Park (TNP), and Lake Manyara National Park (LMNP; Fig 1). The KD is characterized by small-scale and commercial agriculture, interspersed with settlements and remnants of natural vegetation. In the GCA, human settlements and livestock keeping are very prevalent and largely unregulated. In combination with frequent incidences of illegal hunting [47] the resulting mammal community in the GCA is impoverished and densities of most wildlife species are low [44, 48]. MR is a community based conservation entity aimed at balancing needs of pastoral communities and wildlife conservation. Apart from management buildings, no settlements are located within the ranch, livestock keeping is allowed (though with temporal and spatial limitations), and wildlife is protected via regular ranger patrols year-round. Accordingly, the large mammal community is almost intact and several wildlife species occur at relatively high densities [44]. BWMA is multi-use area with specific areas designated for human settlements, livestock grazing, photographic tourism, and hunting. Wildlife populations vary throughout the BWMA based on the land-use plan for each specific area. LMNP and TNP are fully protected national parks where photographic wildlife tourism and research are the only permitted uses. Groundwater forests cover parts of LMNP [49, 50]. In both national parks, regular law enforcement patrols aim to reduce illegal hunting and livestock grazing. Despite experiencing substantial fluctuations in community composition over the past decade, both national parks have high mammal species richness and relatively high population densities of herbivores [51, 52].

### Data collection

Observations in the 6 study areas were conducted along driven line transects during the long rainy season for 10 consecutive days in April 2017, and 9 consecutive days in April 2018. In April 2017, we alternated fieldwork between the 3 study areas, so that at least 48 hours separated sampling events. GCA and LMNP were sampled for 3 separate days and MR was sampled for 4 separate days. In April 2018, we alternated fieldwork between BWMA and TNP so that at least 24 hours separated sampling days, and sampled KD during 3 consecutive days. On all days, sampling started at approximately 8:00 am and ended at approximately 5:00 pm. One mainly continuous transect was chosen each day, ensuring that it was representative of available habitats, and land-uses. Although no transect sections were repeated on a unique day, some sections of transects were repeated in subsequent days, and were partially based on accessibility within each study area (Fig 1). Total transect length across the study area was 886 km (KD = 88 km, GCA = 112.4 km, BWMA = 86.1 km, MR = 178.4 km, LMNP = 172.5 km, TNP = 248.6 km). All ungulates (including African bush elephants; *Loxodonta africana*) spotted within a strip transect of 200 m width were observed by 3 trained researchers for 5 minutes using binoculars, regardless of RBO and YBO presence. All unique RBO and YBO sightings within the transects were documented, including RBOs and YBOs not associated with a host. RBOs were identified by a dark rump, red bill, and yellow orbital ring, while YBOs were identified by a pale rump, bill with yellow base and red tip, and no orbital ring [53]. Mammals were identified to species level, with goats and sheep (*Capra spp*. and *Ovis spp*.) grouped together. Strip half-width was set at 100 m to ensure that all species had a similar chance of being observed [54] and was measured using a laser range finder (Bushnell Elite 1500, Bushnell, Overland Park, KS, USA). If animals within a transect showed signs of nervousness in the presence of our car, we observed animals from up to 200 m (radial distance). Group size (defined as individuals of the same species that were within 50 m of each other), demographics (based on morphology and categorized as: juvenile, adult female, adult male, or unknown), presence of a visible wound, the total number of RBOs and YBOs, and the time of day were recorded for each individual mammal.

During each 5-minute observation conducted in 2018 (KD, BWMA, and TNP), 2 observers sampled RBOs and YBOs quasi-randomly, and documented oxpecker behavior and location on host during a maximum of 6 instantaneous samples separated by 10 s. Potential oxpecker behaviors included wound feeding (oxpeckers feeding on an obvious wound), pecking (repeated pickaxe-like action with bill closed or slightly open, then a pause to collect material, typically used to feed on sores or wounds), plucking (one single pull away from the mammal’s body with a backwards turn of the head), scissoring (the rapid opening and closing of the oxpecker’s mouth as its bill passes over the mammals body or through its hair), insect catching (the oxpecker catches an insect from the air while sitting on the host, or leaves the host to catch an insect before returning), and resting [17]. Possible feeding sites on a mammal included the torso, neck, head, perianal, front legs, hind legs, and underside. Removal attempts and successes by the focal host towards the focal oxpecker were noted continuously.

### Data analysis

#### Landscape-scale distribution

Oxpecker densities were estimated for each day of data collection by dividing the number of observed RBOs and YBOs by the transect area (total line length measured by vehicle odometer multiplied by two times the strip half width of 100 m). Treating each day as a replicate, we calculated 95% confidence intervals and plotted oxpecker density estimates alongside suitable host density estimates (defined as all mammals with body masses between the smallest and largest mammal which RBO and YBO used as hosts) using the R package *gplots* [55, 56]. Body masses of wild mammal species were averaged between the listed value for males and females [57, 58]. Similarly, average cattle (*Bos spp*.), donkey (*Equus africanus*), sheep and goat, and domestic pig (*Sus scrofa domesticus*) body masses were obtained from [59–62] respectively (S1 Appendix). We used a z-test to compare RBO and YBO densities across the study areas [63]. To estimate landscape scale distributions of oxpeckers, we fitted logistic regression models to assess whether RBO and YBO prevalence on individual mammal hosts (presence: 1, absence: 0; irrespective of host species identity) was mediated by study area (fixed effect).

#### Mammal community scale

To assess feeding preferences of RBOs and YBOs at the mammal community scale, we calculated feeding preference indices by dividing the total number of RBOs or YBOs observed on a host species by the total number of observations of that host species [15, 16, 64]. We then fitted a linear regression, testing the RBO and YBO preference indices for each host species across the entire TME against host species body mass. We repeated this regression excluding elephants and hippopotamus (*Hippopotamus amphibius*) because both species represent outliers to oxpecker preference host body mass correlations [16]. Additionally we excluded livestock species to assess their influence on the observed oxpecker preference host body mass correlations. To further illustrate RBO and YBO feeding preferences, we created oxpecker-host species networks using the R package *igraphs* [56, 65], with nodes representing relative host abundance, and edges representing oxpecker abundance on each host species.

#### Host population and individual scale correlates

To assess population and individual level correlates of RBO and YBO preferences, we fitted host and oxpecker species-specific generalized linear models for all species utilized as RBO and YBO hosts (with the exception of hippopotamus and sheep and goat due to low sample size). Models were formulated as fixed-effect logistic regressions (presence/absence of RBOs and YBOs) because RBO and YBO abundance per host was highly over-dispersed and contained excessive zeroes. We did not include the herd identity as mixed effect because within herds there was insufficient variation in the presence and absence of RBOs and YBOs. We considered host demographics (juvenile, adult male, or adult female), time of day (morning defined as 7:30–10:59; midday defined as 11:00–13:59; afternoon defined as 14:00–17:30), group size, and wound presence as fixed effects in a global model. After dredging the global species-specific models (deriving models with all additive explanatory variable permutations), we conducted model selection (S2 Appendix) based on second order Akaike’s information criterion (AICc) using the *MuMIn* package in R [56, 66]. Following recommendations for binomial count data, regression coefficients of all models within Δ-value ≤ 6 were averaged using the zero method [67]. We based our inferences on the effect of explanatory variables on the effect size, associated 95% confidence intervals and relative variable importance.

#### Oxpecker and host behavior

Proportions of oxpecker behaviors were presented for each host species using the program R version 3.5.0 [56]. Differences in host species tolerance were evaluated using a logistic regression, whereas the response variable was defined as a two column object, with scans with removal attempts treated as successes and scans without removal attempts considered failures [68]. Additionally, removal efficiency was presented as the proportion of successful removal attempts for each host species. Species-specific host tolerance (proportion of removal attempts per scan) was correlated with oxpecker host preference indices using Kendall’s correlation test.

## Results

### Landscape-scale distribution

**Fig 2:**
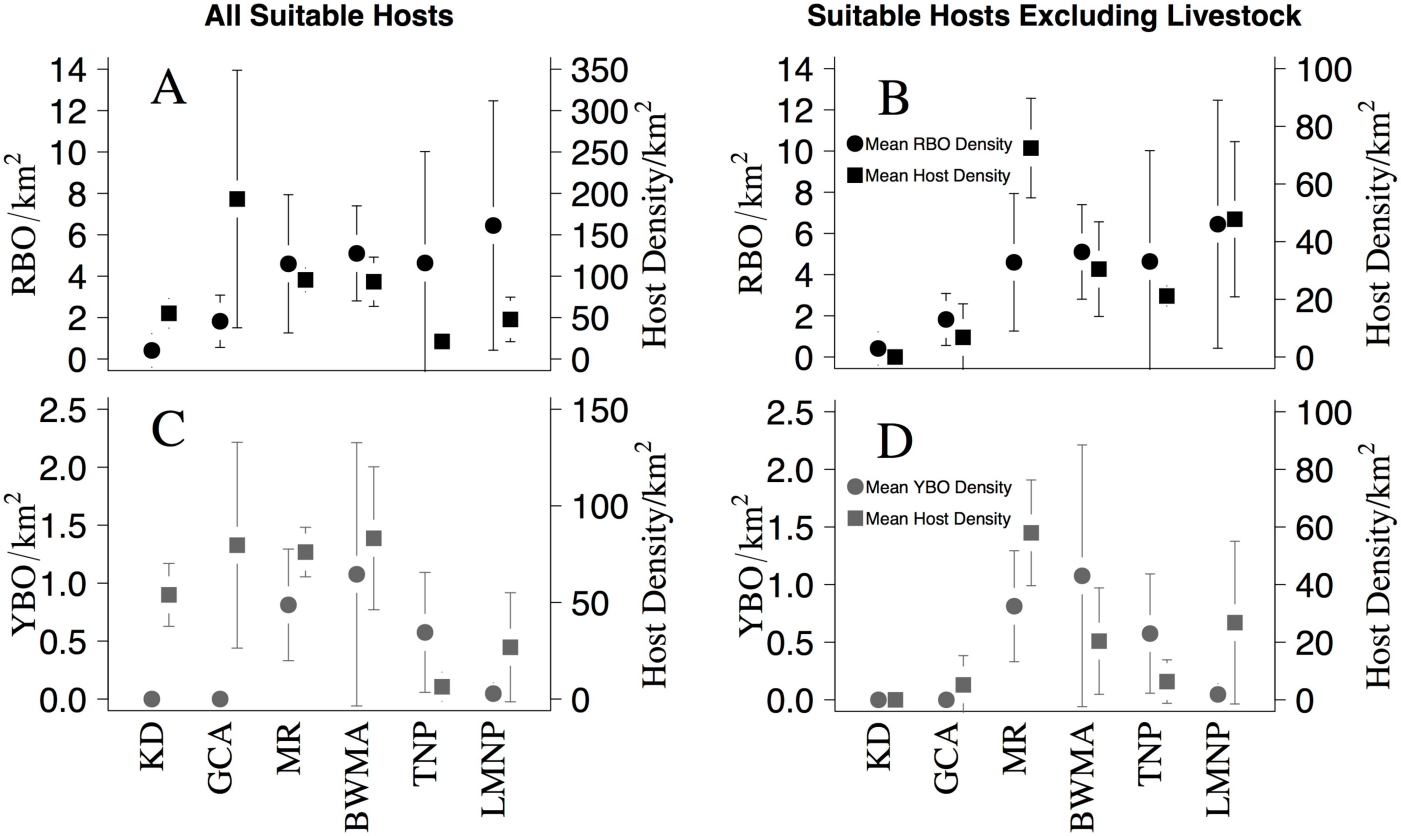
Densities of oxpeckers and suitable hosts across six study areas in northern Tanzania. Red-billed oxpecker (RBO; A, B) and yellow-billed oxpecker (YBO; C, D) densities (incl 95% CI), and average suitable host densities (incl 95% CI) with livestock included as suitable hosts (A, C) and with livestock excluded (B, D). Densities were determined in the Karatu District (KD), Mto Wa Mbu Game Controlled Area (GCA), Manyara Ranch (MR), Burunge Wildlife Management Area (BWMA), Tarangire National Park (TNP) and Lake Manyara National Park (LMNP) within the Tarangire-Manyara ecosystem of northern Tanzania.

Over the entire study period, 705 RBOs, 72 YBOs, and 17,853 individual mammals were observed (S1 Appendix; S3 Appendix). RBO density was lowest in KD and GCA, intermediate in MR, BWMA, and TNP, and highest in LMNP (Fig 2). YBO density followed a similar trend with the exception of the relatively low observed density in LMNP and high density in BWMA (Fig 2). A z-test did not suggest significant differences in RBO densities (all *p*-values > 0.113) and in YBO densities (all p-values > 0.05) across the landscape. However, logistic regression analysis suggested that RBO presence on any available host was significantly higher in BWMA (odds ratio = 7.933), MR (odds ratio = 10.804), TNP (odds ratio = 31.375), and LMNP (odds ratio = 34.467) compared to KD (Table 1). YBO presence (excluding areas without YBO sightings) was significantly higher in MR (odds ratio = 7.698), BWMA (odds ratio = 8.908), and TNP (odds ratio = 22.783) compared to LMNP.

**Table 1:** Summary of logistic regressions testing the effect of study area on oxpecker presence on mammal hosts.

	Red-Billed Oxpecker				
	Estimate	95% CI	Std. Error	*z* value	*p* value
Intercept (KD)	-6.168	-7.338; -5.323	0.500	-12.327	**<0.0001**
GCA	0.545	1.169; 3.271	0.541	1.008	0.314
BWMA	2.071	-0.408; 1.770	0.522	3.968	**<0.0001**
MR	2.380	2.669; 4.725	0.513	4.641	**<0.0001**
TNP	3.446	1.502; 3.568	0.514	6.704	**<0.0001**
LMNP	3.540	2.565; 4.635	0.510	6.936	**<0.0001**
	**Yellow-Billed Oxpecker**				
Intercept (LMNP)	-7.351	-10.215; -5.868	0.999	-7.356	**<0.0001**
BWMA	2.187	0.597; 5.080	1.030	2.123	**0.034**
MR	2.041	0.463; 4.932	1.027	1.988	**0.047**
TNP	3.126	1.547; 6.017	1.027	3.044	**0.002**

Regression coefficients of a binomial logistic regressions testing the effect of study area on red-billed and yellow-billed oxpecker presence across the Karatu District (KD), Mto Wa Mbu Game Controlled Area (GCA), Burunge Wildlife Management Area (BWMA), Manyara Ranch (MR), Tarangire National Park (TNP), and Lake Manyara National Park (LMNP) in the Tarangire-Manyara ecosystem of northern Tanzania. Significant relationships are represented by bold text.

The relative abundance of host species (S3 Appendix) and mean suitable host densities differed across the six study areas, indicating a relatively greater density of potential RBO and YBO hosts in MR, GCA, and BWMA when livestock were included (Fig 2A; Fig 2C). When livestock were considered unsuitable hosts (Fig 2B; Fig 2D), suitable host densities were relatively greater in MR, BWMA, TNP, and LMNP, due to the high relative abundance of livestock (representing 92% of individuals in the mammal community) in GCA when compared to BWMA (36%), MR (23%) and TNP and LMNP (0%; Fig 2). Daily RBO densities were positively and significantly scaled (*F* = 10.4; R^2^ = 0.379; p = 0.005; n = 19 days) with daily suitable host densities when livestock were considered unsuitable hosts, and not correlated (*F* = 0.1; R^2^ = 0.006; p = 0.752; n = 19) with suitable host densities when livestock were included as suitable hosts. Similarly, daily YBO densities showed a weak positive correlation (*F* = 2.2; R^2^ = 0.115; p = 0.155; n = 19) with suitable host densities excluding livestock, and no correlation (*F* = 0.1; R^2^ = 0.005; p = 0.767; n = 19) when livestock were considered suitable hosts.

### Host-community scale correlates

**Fig 3:**
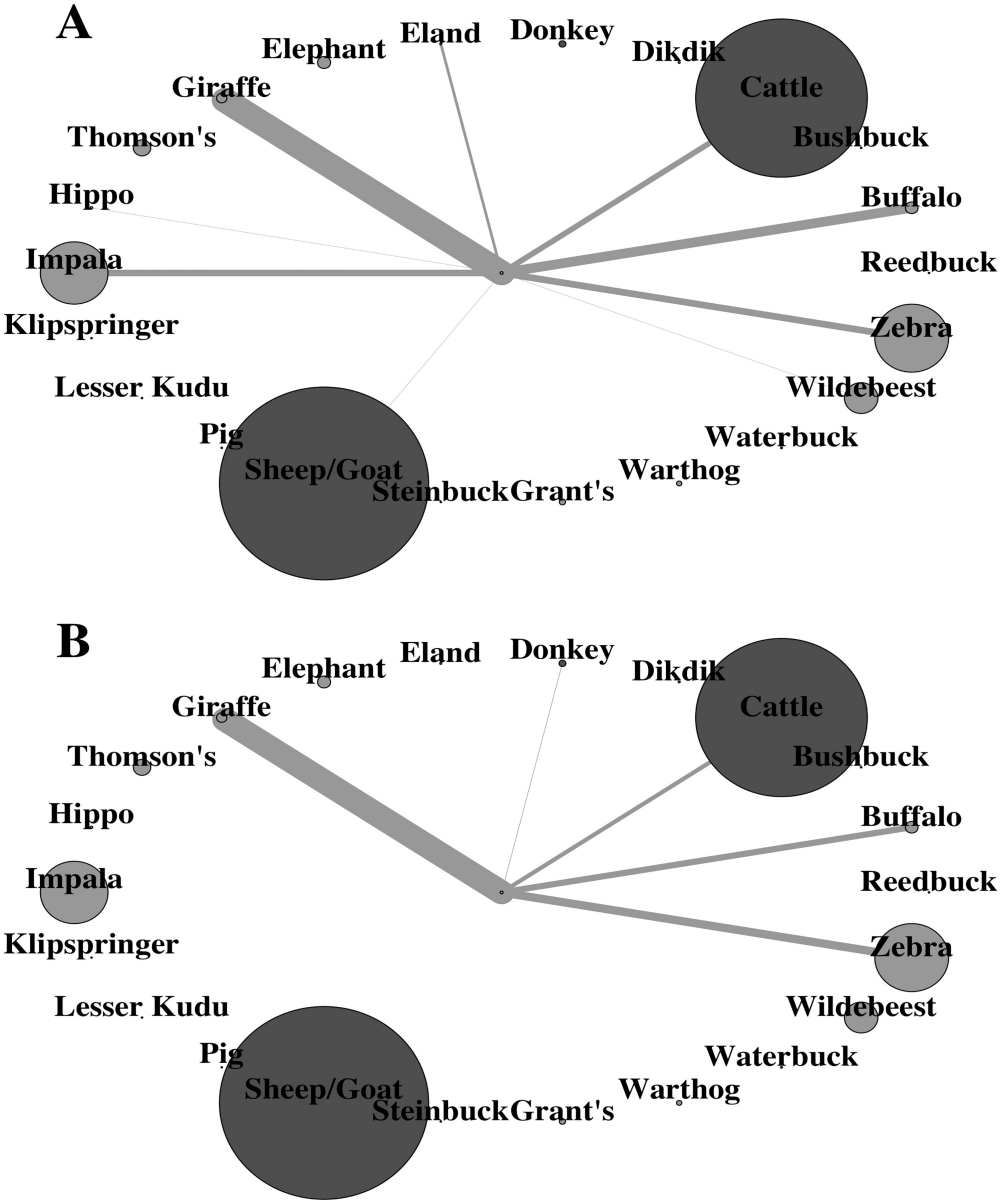
Networks illustrating oxpecker host species preferences. Visual representations of the red-billed oxpecker (A) and yellow-billed oxpecker (B) mammal host networks in the Tarangire-Manyara ecosystem of northern Tanzania. Nodes represent host species abundance and edges represent total oxpeckers observed on each host species across all study areas.

Overall, RBOs were observed on 6 host species in LMNP, 5 in MR and BWMA, 3 in TNP, and 1 in GCA and KD (S3 Appendix). YBOs were observed on 4 host species in BWMA, 2 in MR and TNP, and 1 in LMNP (Fig 3; S1 Appendix). Across the TME, RBO and YBO preferences showed a strong positive correlation with host species body masses when hippopotamus and elephants were excluded, highlighting RBO and YBO preference for large mammals up to a threshold; 67% of all RBO and 70% YBO observed on mammals weighing between 500 and 1500kg: buffalo, giraffe, and eland (Table 2; Fig 3).

**Table 2:** Percentages of oxpeckers observed on a specific host species across six study areas in northern Tanzania.

Oxpecker Species		KD	GCA	BWMA	MR	LMNP	TNP
**RBO**	Buffalo					58.5%	3.3%
	Cattle	100%	100%	20.7%			
	Eland				19.6%		
	Giraffe			59.8%	52.3%	10.9%	73.3%
	Hippopotamus					0.5%	
	Impala			5.4%	6.5%	10.4%	23.3%
	Sheep/Goat			2.2%			
	Wildebeest				0.7%	0.5%	
	Zebra			12%	20.9%	19.1%	
**YBO**	Buffalo					100%	33.3%
	Cattle			38.1%			
	Donkey			4.8%			
	Giraffe			52.4%	46.4%		66.7%
	Zebra			4.8%	53.6%		

Percentages of red-billed (RBO) and yellow-billed (YBO) oxpeckers observed on mammal species within the Karatu District (KD) Mto Wa Mbu Game Controlled Area (GCA), Burunge Wildlife Management Area (BWMA), Manyara Ranch (MR), Tarangire National Park (TNP) and Lake Manyara National Park (LMNP) in the Tarangire-Manyara ecosystem of northern Tanzania.

**Fig 4:**
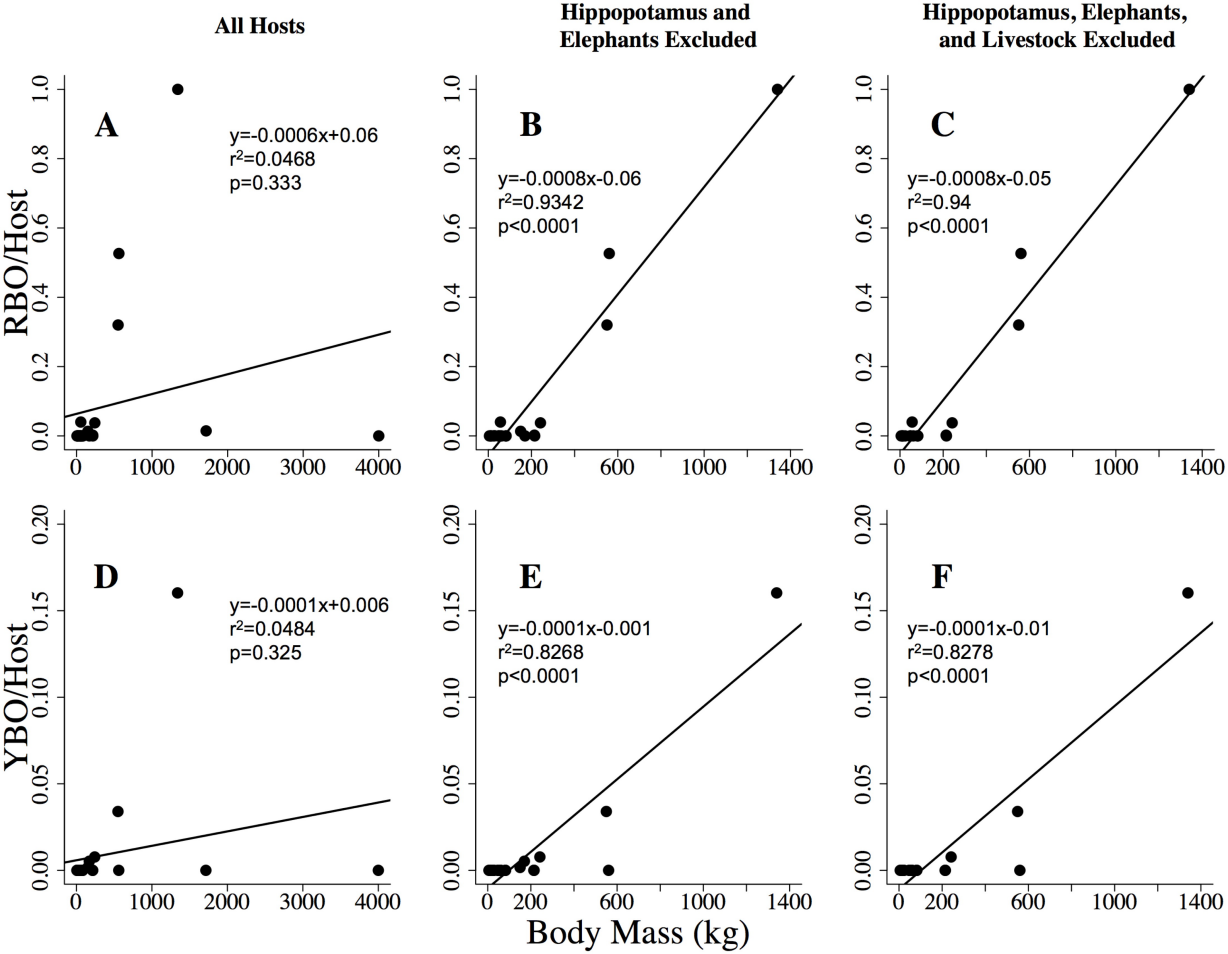
Linear regressions of oxpecker preference indices with host species body masses. Linear regressions between red-billed (RBO) and yellow-billed (YBO) oxpecker preference indices and host body masses based on different definitions of suitable host species: considering all mammal hosts (left panel), excluding hippopotamus and elephant (center panel) and excluding hippopotamus, elephant and livestock (right panel). Observations were conducted across six study areas in the Tarangire-Manyara Ecosystem of northern Tanzania. Points represent mammal host species. Body masses were obtained from values listed in the literature (S1 Appendix).

Across the sampled areas, RBO and YBO preferences were significantly (RBO: *F* = 255.7; *p* <0.0001; *df* = 18; YBO: *F* = 85.92; *p* <0.0001; *df* = 18) correlated with host species body masses when elephants and hippopotamus were excluded, which explained 93% and 83% (*R*^*2*^) of the variability in the host preference indices respectively (Fig 4). When elephants and hippopotamus were included as suitable hosts, body mass did not explain the variability in RBO preferences (*F* = 0.98; *R*^*2*^ = 0.05 *p* = 0.334; *df* = 20), or YBO preferences (*F* = 1.01; *R*^*2*^ = 0.05 *p* = 0.325; *df* = 20). *R*^2^ values increased slightly (in RBO from 93% to 94%, in YBO from 82.7% to 82.8%) when livestock species were excluded from the linear regressions.

### Population and individual scale correlates

Model selection indicated that host demographics, group size, time of day, and wound presence were important factors influencing RBO and YBO presence across all host species with adequate sample sizes. Based on relative variable importance, host demography was a key variable explaining RBO and YBO presence on buffalo, cattle, giraffe, and eland (RBO only). Male individuals were typically more frequented by RBOs and YBOs compared to females, however, several confidence intervals overlapped zero (Table 3). Similarly, juvenile individuals were less likely to be utilized as hosts by RBOs when compared to adults with the exception of wildebeest, and less likely to be utilized by YBOs with the exception of zebra. Time of day was an inconsistent factor influencing RBO and YBO presence for all host species. Wound presence generally increased the presence of RBO on a host species, but usually decreased YBO presence (except for giraffe); however, relative variable importance of wound presence was comparatively low. For most host species, there was a negative trend for the relationship between host group size and RBO presence on individual hosts with the exception of impala, whereas the group size YBO presence relationship showed the opposite trend.

**Table 3:** Summary of logistic regression models explaining oxpecker presence on large mammal species as a function of individual and population scale variables.

**Red-Billed Oxpecker**									
Species		Intercept	Demographics			Time of Day		Wound Presence	Group size
			Juvenile	Male	Unknown	Morning	Midday		
Buffalo	Est.	-1.772	-1.102	0.487	-17.920	1.518	-18.410	0.152	-0.331
	CI	-2.931; -0.613	-1.877; -0.326	-0.264; 1.237	-1761; 1725	0.390; 2.646	-7066; 7030	-1.206; 1.286	-0.645; 0.418
	SE	0.591	0.396	0.383	889	0.576	3596	1.235	0.378
	RVI		1.00	1.00	1.00	1.00	1.00	0.26	0.34
Cattle	Est.	-4.087	-0.708	1.377	0.007	-1.279	-1.203	0.585	-0.484
	CI	-4.796; -3.378	-1.676; 0.260	0.561; 2.191	-0.889; 0.903	-2.073; -0.495	-1.980; -0.426	-0.983; 2.572	-1.448; 0.479
	SE	0.362	0.494	0.416	0.457	0.405	0.397	0.800	0.491
	RVI		1.00	1.00	1.00	1.00	1.00	0.49	0.65
Eland	Est.	-1.669	-15.432	1.277		-15.797	1.829	6.359	-1.507
	CI	-198; 194	-5643; 5612	-0.827; 3.382		-5526; 5495	-1.515; 5.173	-11190; 11210	-5.148; 2.134
	SE	100	2871	1.074		2812	1.706	5714	1.858
	RVI		0.85	0.85		0.88	0.88	0.34	0.62
Giraffe	Est.	-0.429	-0.263	0.152	-14.344	0.002	-0.010	0.581	-0.744
	CI	-0.932; 0.074	-0.888; 0.362	-0.442; 0.746	-1336; 1307	-0.220; 0.224	-0.211; 0.191	-0.617; 1.779	-1.959; 0.470
	SE	0.257	0.329	0.303	674	0.113	0.103	0.611	0.619
	RVI		0.87	0.87	0.87	0.10	0.10	0.64	0.76
Impala	Est.	-3.323	-0.058	-0.063	-2.291	-0.050	-0.116	-3.823	0.104
	CI	-6.404; -0.242	-0.415; 0.299	-0.463; 0.337	-523; 518	-0.391; 0.292	-0.625; 0.393	-832; 825	-0.302; 0.510
	SE	1.572	0.182	0.204	226	0.174	0.260	423.0	0.207
	RVI		0.17	0.17	0.17	0.25	0.25	0.31	0.38
Wildebeest	Est.	-21.677	0.019	2.842	0.037	2.922	2.936	3.568	-20.311
	CI	-4338; 4296	-5231; 5231	-3550; 3555	-8425; 8425	-2451; 2457	-2451; 2457	-32488; 32495	-53.536; 12.913
	SE	2203	2669	1812	4299	1252	1252	16580	16.950
	RVI		0.15	0.15	0.15	0.17	0.17	0.25	0.90
Zebra	Est.	-3.966	-0.050	0.010	-0.016	-0.129	-0.065	0.100	-0.049
	CI	-4.408; -3.522	-0.488; 0.348	-0.288; 0.308	-0.325; 0.293	-0.765; 0.506	-0.488; 0.358	-1.003; 1.202	-0.485; 0.360
	SE	0.226	0.203	0.152	0.157	0.324	0.216	0.563	0.209
	RVI		0.09	0.09	0.09	0.22	0.22	0.27	0.29
**Yellow-Billed Oxpecker**									
Species		Intercept	Demographics			Time of Day		Wound Presence	Group size
			Juvenile	Male	Unknown	Morning	Midday		
Buffalo	Est.	-0.461	-20.234	0.236	-23.163	-4.230	-19.150	-13.278	2.952
	CI	-342; 341	-9605; 9564	-1.817; 2.289	-7519; 7473	-6.358; -2.103	-31080; 31040	-24180; 24160	-0.724; 5.179
	SE	174	4890	1.048	3825	1.086	15850	12330	1.137
	RVI		1.00	1.00	1.00	1.00	1.00	0.61	1.00
Cattle	Est.	-18.355	-15.495	3.330	-16.930	-0.157	10.129	-6.686	2.316
	CI	-5081; 5044	-4894; 4863	1.217; 5.442	-5660; 5627	-6598; 6497	-5749; 5070	-11162; 11149	0.528; 4.104
	SE	2583	2489	1.078	2879	3315	2581	5692	0.912
	RVI		1.00	1.00	1.00	0.58	0.58	0.36	1.00
Giraffe	Est.	-2.391	-0.559	0.632	-14.626	0.032	-0.001	0.832	0.024
	CI	-3.159; -1.624	-1.809; 0.691	-0.363; 1.627	-2243; 2214	-0.396; 0.461	-0.361; 0.342	-0.643; 2.308	-0.670; 0.718
	SE	0.392	0.638	0.508	1137	0.219	0.179	0.753	0.354
	RVI		0.90	0.90	0.90	0.11	0.11	0.69	0.28
Zebra	Est.	-6.787	0.124	0.154	0.132	2.447	0.507	-4.298	0.339
	CI	-40.205; 26.630	-0.746; 0.994	-0.909; 1.216	-0.853; 1.118	0.333; 4.560	-1.941; 2.954	-1731; 1723	-0.910; 1.587
	SE	17.050	0.444	0.542	0.503	1.079	1.249	881	0.637
	RVI		0.12	0.12	0.12	1.00	1.00	0.30	0.39

Regression coefficients (and associated statistics) for individual level variables influencing red-billed and yellow-billed oxpecker associations with large mammal species. Separate negative binomial regressions were created for each host species, and models with ΔAICc-values ≤ 6 were combined using the full average method (S1 Appendix) [67]. For values >100 no decimals were displayed.

### Oxpecker and host behavior

**Fig 5:**
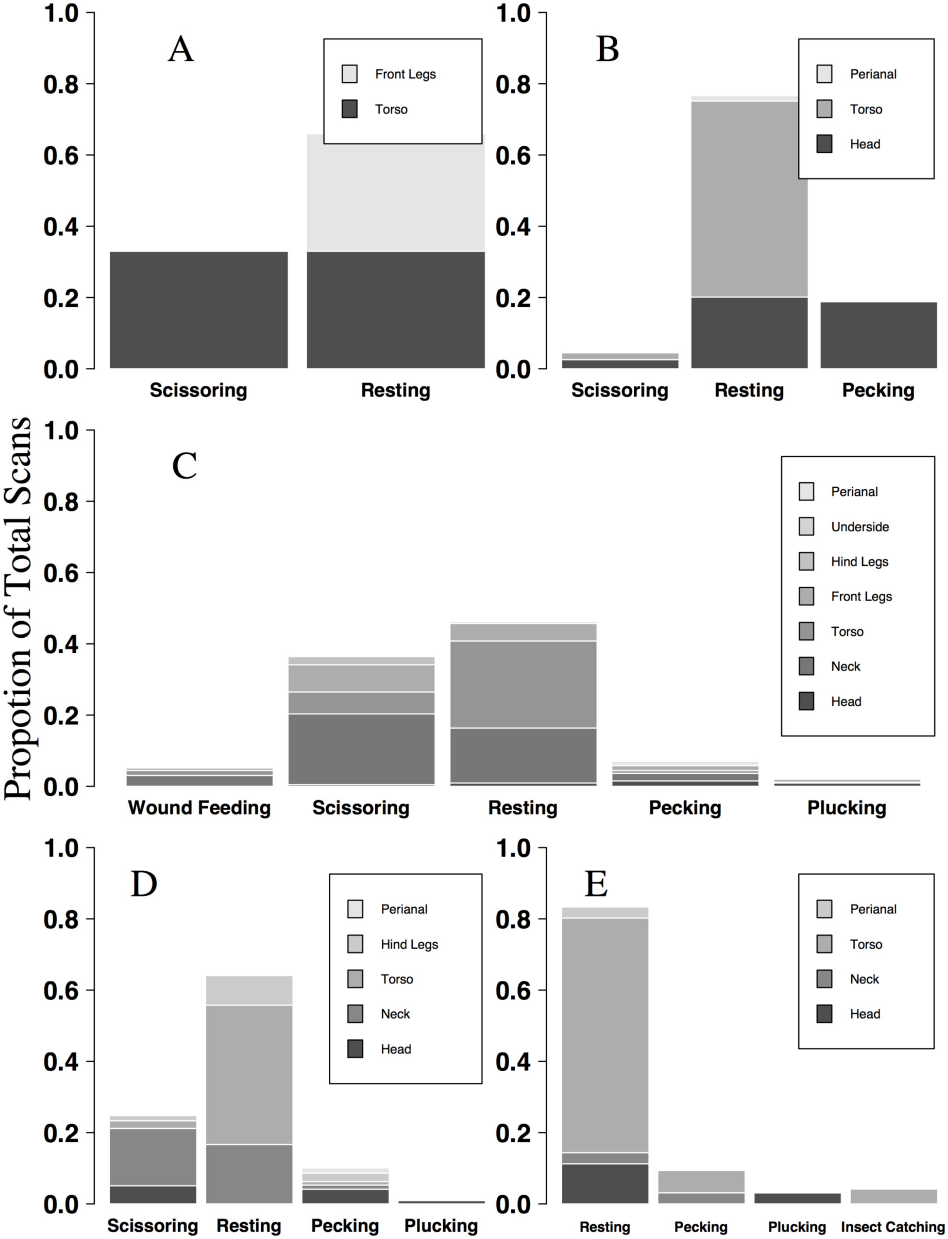
Proportion of red-billed oxpecker behaviors observed on five host species in northern Tanzania. Behaviors and attachment sites of individual red-billed oxpeckers were instantaneously sampled while birds were associated with buffalo (A; n = 3 observations), cattle (B; n = 13 observations), giraffe (C; n = 71 observations), impala (D; n = 17 observations) and zebra (E; n = 17 observations) across three study areas in the Tarangire-Manayara ecosystem of northern Tanzania.

**Fig 6:**
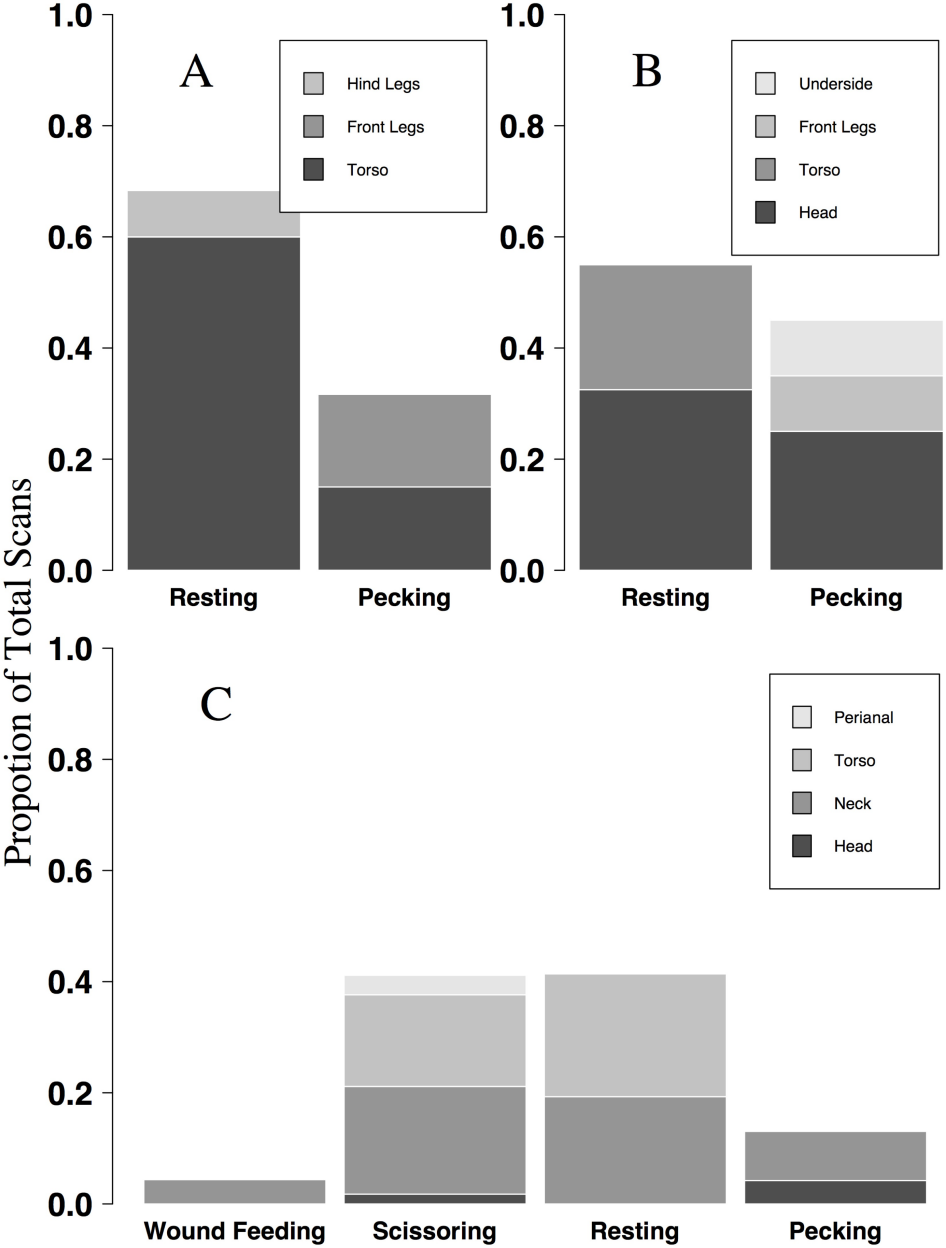
Proportion of yellow-billed oxpecker behaviors observed on three host species in northern Tanzania. Behaviors and attachment sites of individual yellow-billed oxpeckers were instantaneously sampled while birds were associated with buffalo (A; n = 4 observations), cattle (B; n = 2 observations), giraffe (C; n = 19 observations), across three study areas in the Tarangire-Manayara ecosystem of northern Tanzania.

Resting and scissoring (typically on the torso and neck) comprised more than 80% of observed RBO behaviors when associated with buffalo, cattle, giraffe, and impala hosts (Fig 5). When associated with giraffe, patterns of YBO behavior and attachment locations were similar to those of the RBO (Fig 6C). In addition, resting constituted 40–70% of YBO behavior across buffalo, cattle, and giraffe. Contrary to RBOs, YBOs were never observed scissoring on buffalo and cattle, and pecking comprised 30–45% of YBO behavior on these hosts (Fig 6). Wound feeding by both RBO and YBO was only observed on giraffe, and comprised 6% and 4% of total observed behavior on giraffe respectively.

**Fig 7:**
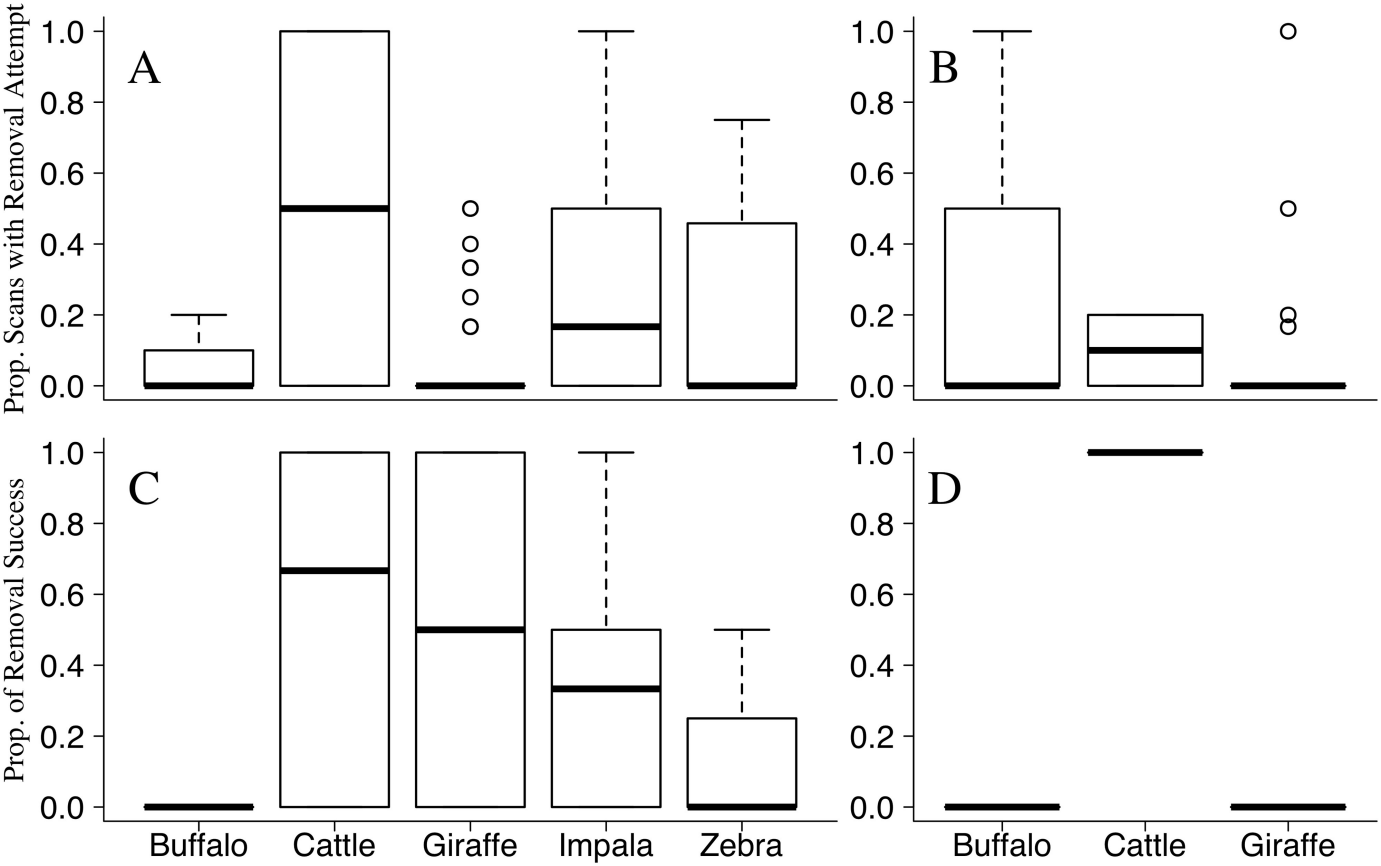
Host species tolerance of oxpeckers and host removal efficiency. Attempts by a host individual to remove an associated focal red-billed (A) or yellow-billed (B) oxpecker during instantaneous scans were averaged for each host species. Successful removal attempts are displayed as proportion of successful removals per removal attempt for each host species associated with red-billed (C) and yellow-billed (D) oxpeckers.

Host attempts to remove RBOs were significantly more common among cattle (odds ratio = 13.518), impala (odds ratio = 8.029), and zebra (odds ratio = 4.371) when compared to giraffe (Table 4; Fig 7A). Cattle were most successful in removing RBOs, followed by giraffe, impala, zebra and buffalo (Fig 7C). Similarly, attempts to remove associated YBOs were most commonly observed among cattle when compared to giraffe and buffalo (Fig 7B), and all cattle attempts were successful in removing YBOs (Fig 7D). Considering the small sample size, host tolerance (the proportion of scans where a host species attempted to remove an oxpecker) appeared to be negatively correlated with oxpecker preference for that host species (RBO τ = -0.8, n = 5, p = 0.083; YBO τ = -0.33, n = 3, p = 1).

**Table 4:** Summary statistics of a logistic regression testing the likelihood of attempts by a host species to remove associated red-billed oxpeckers.

	Estimate	95% CI	Std. Error	*z* value	*p* value
Intercept (Giraffe)	-2.979	-3.542; -2.497	0.265	-11.255	**<0.0001**
Buffalo	0.782	-2.165; 2.550	1.087	0.719	0.472
Cattle	2.604	1.670; 3.538	0.473	5.509	**<0.0001**
Impala	2.083	1.354; 2.833	0.375	5.557	**<0.0001**
Zebra	1.475	0.379; 2.464	0.523	2.819	**0.005**

The logistic regression tested the likelihood of a host species attempting to remove a red-billed oxpecker. Scans with removal attempts were considered successes and scans without removal attempts were considered failures. Significant relationships are represented by bold text.

## Discussion

Our findings tie the decline of mutualistic mammal-bird interactions with the loss of wild, large bodied (between 500 and 1500 kg) herbivore populations. The observed variation in landscape-scale oxpecker density appears to be linked with specialized oxpecker preferences for large mammal host species, and for large individuals within a given species. This further supports the ecological importance of large wild herbivore populations in savanna ecosystems, and provides insight into the complex interactions between large mammals, and associated symbionts [1, 13, 69]. Ultimately, the oxpecker large mammal associations represent excellent contemporary model systems for investigating the coextinction vulnerability of large mammal bird mutualisms [1, 13].

### Why do oxpeckers prefer large-bodied hosts?

Oxpecker preferences for large (between 500 kg and 1500kg) mammals are likely due to (1) the relatively high ectoparasite abundance harbored by large bodied hosts [22, 24, 25], and (2) a high, likely coevolved tolerance towards foraging oxpeckers exhibited by most, but not all large mammal species [27–29]. Similar to previous studies, YBOs showed a narrower range of preferred hosts compared to RBOs, which may be due to their higher energy demands resulting from YBOs larger body mass relative to the RBO [15, 70]. While additional factors may influence oxpecker host preferences, forage abundance has been demonstrated to be a principle driver of third order habitat selection (in this case oxpecker selection of hosts within a community) throughout the animal kingdom [71, 72]. In line with overall oxpecker preferences for relative large host species and individuals (adults and males), and weak evidence for oxpecker parasitism (indicated by the non-significant effect of wound presence on oxpecker feeding and the limited amount of observed wound feeding), oxpecker preferences for large bodied ungulate hosts appear to be partially a function of available ectoparasite forage abundance [15]. Wallowing by African elephants and common hippopotamus, which effectively reduces ectoparasite abundance [73] and hippopotamus being partially submerged in water during daytime, may partially explain why these two species deviate from the observed oxpecker preferences for large bodied hosts [16].

Previous research has demonstrated that large mammals exhibit a higher tolerance for oxpeckers relative to smaller mammals, which may be due to reduced agility of large-bodied hosts and/or the coevolution of a mutualism [27–29]. We show that cattle, impala, and zebra are less tolerant of oxpecker presence when compared to highly preferred giraffe and buffalo. In addition, we show that giraffe can remove RBOs efficiently, indicating that tolerance is not strictly the result of reduced agility due to host body size [27–29]. Cattle removed oxpeckers efficiently, and in 93% of all behavior observations on cattle, oxpeckers left host individuals before a full minute observation could be completed (as opposed to 53% for impala, 38% for zebra, 43% for buffalo and 27% for giraffe). In accordance with optimal foraging theory [38, 39], the availability of a stable feeding platform reduces oxpecker-feeding effort (e.g. less effort spent switching hosts, high energy gain on a single host), which may further explain oxpecker preferences for tolerant ungulate species.

Overall, our findings suggest that oxpecker tolerance is host species specific. Relatively low oxpecker preferences for cattle may primarily be the result of cattle behavior, which frequently and effectively removes oxpeckers. Variability in host tolerance may be due to differences in the coevolutionary history of host species and oxpeckers, since livestock species were only introduced to African savannahs approximately 5000–7000 years ago [74]. An alternative, mutually non-exclusive hypothesis may be that for certain host species (i.e. giraffe, buffalo, eland) oxpecker feeding is primarily mutualistic; while for other host species (i.e. cattle) feeding is potentially more parasitic. Overall, these results strongly suggest that oxpecker-host relationships differ markedly across host species and that evaluating these relationships (e.g. commensal, mutualistic, parasitic, or opportunistic parasitism) requires a more nuanced, multi-species approach [19, 21, 75].

### Are we witnessing coextinctions?

Several hypothesized underlying mechanisms contributing to oxpecker declines in southern Africa have been suggested, including unsuitable climate and climatic change [18, 30, 31], oxpecker mortality caused by arsenic acaricides [17], reduced individual host and landscape-scale tick abundance due to acaricide treatment [76], and low livestock tolerance towards oxpeckers [40, 75, 77]. While the effects of acaricides on landscape-scale tick abundance have not been assessed in our study area, acaricides currently used in Tanzania do not contain arsenic [78], and previous research suggests that tick burden on livestock in our study area is high [79, 80] despite frequent use of acaricides [78]. Despite little evidence about the direct effect of acaricides on landscape-scale tick and oxpecker abundance, it is a plausible hypothesis that warrants further investigation.

Paleontological studies suggest that megafauna extinctions during the Late Pleistocene and early Holocene led to subsequent coextinction cascades of commensalistic bird species that were incapable of adaptive shifting [1, 81]. Indeed, oxpeckers have undergone substantial range reductions and local extinctions in parts of South Africa, following the replacement of preferred large-bodied wild ungulate hosts with livestock [18, 30, 82]. Globally, large mammals have either gone locally extinct or have undergone substantial population declines [83, 84], a pattern that is mirrored within protected areas in the Tarangire-Manyara ecosystem [51, 52], and especially pronounced in areas with little or no protection [32].

By assessing the potential for adaptive host shifting, and the resulting fitness effects on oxpeckers, we can better predict the consequences of ongoing defaunation in African savanna ecosystems for this commensalism. In line with previous research, we show that oxpeckers (mainly RBOs) utilize relatively small host species to some extent, especially impala, zebra [15, 16, 27], and cattle [21]. While some of these wild ungulate host species persist in human dominated landscapes, with cattle occurring at very high densities [44], our data suggest that these relatively small host species can support a substantially smaller RBO population density. Most likely, this results from the low feeding efficiency associated with utilizing small wild-ungulate hosts and livestock species caused by two principal mechanisms: low host tolerance (particularly observed in cattle), and relatively low ectoparasite abundance [22].

Overall, these considerations suggest that host-preference plasticity (particularly in RBOs) may prevent oxpecker coextinctions in human-dominated landscapes to some extent, particularly if large wild herbivores are effectively conserved in adjacent protected areas, which may serve as source populations for oxpeckers. However, following the small population paradigm [85], the resulting sparser oxpecker populations may be subject to stochasticity, which may lead to time-lagged local extinctions of oxpeckers [18, 86]. Although livestock in our study area may have high tick burdens [79], livestock were rarely utilized by oxpeckers. Low tolerance of the livestock towards oxpeckers as well as herder intervention to discourage oxpecker feeding (although possible, we did not observe herder intervention during the study) may lower the apparent profitability of livestock as host species for oxpeckers.

## Conclusion

We show that defaunation, particularly declines of large ungulates between 500 and 1500 kg, is most likely the key reason for reduced densities of RBOs and YBO in human- and livestock-dominated East African savannas. While available as potential hosts, livestock (especially cattle) have a relatively low tolerance for oxpecker attachment and feeding and therefore cannot effectively replace more preferred and suitable wild mammal hosts. As such, declines in large mammal populations likely cause declines in these two mutualistic bird species, which may have further cascading effects.

## Supporting information

S1 Appendix Total number of red and yellow-billed oxpeckers observed associated with each host species across six study areas in northern Tanzania. All oxpeckers observed on a host were recorded across 3 days of data collection in the Karatu District (KD), Burunge Wildlife Management Area (BWMA), Tarangire National Park (TNP), Mto wa Mbu Game Controlled Area (GCA), and Lake Manyara National Park (LMNP), and 4 days of data collection in Manyara Ranch (MR).
(DOCX)

S2 Appendix Model selection tables for host-specific logistic regression models explaining red and yellow-billed oxpecker (RBO and YBO respectively) presence in relation to individual and population scale correlates. (DOCX)

S3 Appendix Total number of individual hosts observed and host species relative abundance across six study areas in northern Tanzania. All host individuals were recorded across 3 days of data collection in the Karatu District (KD), Burunge Wildlife Management Area (BWMA), Tarangire National Park (TNP), Mto wa Mbu Game Controlled Area (GCA), and 4 days of data collection in Manyara Ranch (MR).
(DOCX)

## References

[1] Galetti M, Moleón M, Jordano P, Pires MM, Guimarães PR, Pape T, et al Ecological and evolutionary legacy of megafauna extinctions. Biological Reviews. 2018;93:845–62. 10.1111/brv.12374 28990321

[2] Estes JA, Terborgh J, Brashares JS, Power ME, Berger J, Bond WJ, et al Trophic downgrading of planet Earth. Science. 2011;333:301–6. 10.1126/science.1205106 21764740

[3] Ripple WJ, Newsome TM, Wolf C, Dirzo R, Everatt KT, Galetti M, et al Collapse of the world's largest herbivores. Science advances. 2015;1:e1400103 10.1126/sciadv.1400103 26601172 PMC4640652

[4] Craigie ID, Baillie JEM, Balmford A, Carbone C, Collen B, Green RE, et al Large mammal population declines in Africa's protected areas. Biological Conservation. 2010;143:2221–8.

[5] Brashares JS, Gaynor KM. Eating ecosystems. Science. 2017;356:136–7. 10.1126/science.aan0499 28408560

[6] Buckley RC, Castley JG, de Pegas FV, Mossaz AC, Steven R. A population accounting approach to assess tourism contributions to conservation of IUCN-redlisted mammal species. PLoS ONE. 2012;7:e44134 10.1371/journal.pone.0044134 22984467 PMC3440393

[7] Montoya JM, Donohue I, Pimm SL. Planetary boundaries for biodiversity: implausible science, pernicious policies. Trends in Ecology & Evolution. 2017;33:71–3. 29126565 10.1016/j.tree.2017.10.004

[8] Chritz KL, Blumenthal SA, Cerling TE, Klingel H. Hippopotamus (*H*. *amphibius*) diet change indicates herbaceous plant encroachment following megaherbivore population collapse. Scientific reports. 2016;6:32807 10.1038/srep32807 27616433 PMC5018729

[9] Beaune D, Fruth B, Bollache L, Hohmann G, Bretagnolle F. Doom of the elephant-dependent trees in a Congo tropical forest. Forest Ecology and Management. 2013;295:109–17.

[10] Cochrane EP. The need to be eaten: *Balanites wilsoniana* with and without elephant seed-dispersal. Journal of Tropical Ecology. 2003;19:579–89.

[11] Steadman DW, Martin PS. The late Quaternary extinction and future resurrection of birds on Pacific islands. Earth Science Reviews. 2003;61:133–47.

[12] Colwell RK, Dunn RR, Harris NC. Coextinction and persistence of dependent species in a changing world. Annual Review of Ecology, Evolution, and Systematics. 2012;43:183–203.

[13] Dunn RR, Harris NC, Colwell RK, Koh LP, Sodhi NS. The sixth mass coextinction: are most endangered species parasites and mutualists? Proceedings of the Royal Society B: Biological Sciences. 2009;276:3037–45. 10.1098/rspb.2009.0413 19474041 PMC2817118

[14] Mikula P, Hadrava J, Albrecht T, Tryjanowski P. Large-scale assessment of commensalistic-mutualistic associations between African birds and herbivorous mammals using internet photos. PeerJ. 2018;6:e4520 10.7717/peerj.4520 29576981 PMC5863707

[15] Nunn CL, Ezenwa VO, Arnold C, Koenig WD. Mutualism or parasitism? Using a phylogenetic approach to characterize the oxpecker-ungulate relationship: opecker host preferences. Evolution. 2011;65:1297–304. 21166791 10.1111/j.1558-5646.2010.01212.x

[16] Koenig WD. Host preferences and behaviour of oxpeckers: co-existence of similar species in a fragmented landscape. Evolutionary Ecology. 1997;11:91–104.

[17] Bezuidenhout JD, Stutterheim CJ. A critical evaluation of the role played by the red-billed oxpecker *Buphagus erythrorhynchus* in the biological control of ticks. Onderstepoort Journal of Veterinary Research 1980;47:51–75. 7413164

[18] Stutterheim C, Brooke R. Past and present ecological distribution of the yellowbilled oxpecker in South Africa. South African Journal of Zoology. 1981;16:44–9.

[19] Plantan T, Howitt M, Kotzé A, Gaines M. Feeding preferences of the red‐billed oxpecker, *Buphagus erythrorhynchus*: a parasitic mutualist? African Journal of Ecology. 2012;51:325–36.

[20] Plantan TB. Feeding behavior of wild and captive oxpeckers (Buphagus spp.): A case of conditional mutualism: PhD thesis, Univeristy of Miami, Coral Gables, Florida; 2009.

[21] Weeks P. Red-billed oxpeckers: vampires or tickbirds? Behavioral Ecology. 2000;11:154–60.

[22] Esser HJ, Foley JE, Bongers F, Herre EA, Miller MJ, Prins HHT, et al Host body size and the diversity of tick assemblages on Neotropical vertebrates. International Journal for Parasitology: Parasites and Wildlife. 2016;5:295–304. 10.1016/j.ijppaw.2016.10.001 27812506 PMC5078680

[23] Gallivan GJ, Horak IG. Body size and habitat as determinants of tick infestations of wild ungulates in South Africa. South African Journal of Wildlife Research. 1997;27:63–70.

[24] Poulin R, George-Nascimento M. The scaling of total parasite biomass with host body mass. International Journal for Parasitology. 2007;37:359–64. 10.1016/j.ijpara.2006.11.009 17196596

[25] Kiffner C, Stanko M, Morand S, Khokhlova IS, Shenbrot GI, Laudisoit A, et al Sex-biased parasitism is not universal: evidence from rodent—flea associations from three biomes. Oecologia. 2013;173:1009–22. 10.1007/s00442-013-2664-1 23636459

[26] Kiffner C, Lödige C, Alings M, Vor T, Rühe F. Body‐mass or sex‐biased tick parasitism in roe deer (*Capreolus capreolus*)? A GAMLSS approach. Medical and Veterinary Entomology. 2011;25:39–45. 10.1111/j.1365-2915.2010.00929.x 21118286

[27] Ndlovu M, Combrink L. Feeding preferences of oxpeckers in Kruger National Park, South Africa. Koedoe. 2015;57:1–6.

[28] Grobler JH. Host selection and species preference of the red-billed oxpecker *Buphagus erythrorhynchus* in the Kruger National Park. Koedoe. 1980;23:89–97.

[29] Hustler K. Host preference of oxpeckers in the Hwange National Park, Zimbabwe. African Journal of Ecology. 1987;25:241–5.

[30] Stutterheim CJ. Past and present ecological distribution of the redbilled oxpecker (*Buphagus erythrorhynchus)* in South Africa (*Rhipicephalus appendiculatus*). South African Journal of Zoology. 1982;17:190–6.

[31] Robertson A, Jarvis AM. Oxpeckers in north-eastern Namibia: recent population trends and the possible negative impacts of drought and fire. Biological Conservation. 2000;92:241–7.

[32] Kiffner C, Wenner C, LaViolet A, Yeh K, Kioko J. From savannah to farmland: effects of land‐use on mammal communities in the Tarangire–Manyara ecosystem, Tanzania. African Journal of Ecology. 2015;53:156–66.

[33] Manly BFJ. Resource Selection by Animals: Statistical Design and Analysis for Field Studies. Second ed Dordrecht: Kluwer Academic Publishers; 2002.

[34] Hanya G, Chapman CA. Linking feeding ecology and population abundance: a review of food resource limitation on primates. Ecological Research. 2013;28:183–90.

[35] Boyce MS, McDonald LL. Relating populations to habitats using resource selection functions. Trends in Ecology & Evolution. 1999;14:268–72. 10370262 10.1016/s0169-5347(99)01593-1

[36] Bencin H, Kioko J, Kiffner C. Local people’s perceptions of wildlife species in two distinct landscapes of Northern Tanzania. Journal for Nature Conservation. 2016;34:82–92.

[37] Mooring MS, Benjamin JE, Harte CR, Herzog NB. Testing the interspecific body size principle in ungulates: the smaller they come, the harder they groom. Animal Behaviour. 2000;60:35–45. 10.1006/anbe.2000.1461 10924201

[38] MacArthur RH, Pianka ER. On optimal use of a patchy environment. American Naturalist. 1966;100:603–9.

[39] Pyke GH, Pulliam HR, Charnov EL. Optimal foraging: a selective review of theory and tests. Quarterly Review of Biology. 1977;52:137–54.

[40] Attwell RIG. Oxpeckers, and their associations with mammals in Zambia. Puku. 1966;4:17–48.

[41] Kiffner C, Vor T, Hagedorn P, Niedrig M, Rühe F. Factors affecting patterns of tick parasitism on forest rodents in tick-borne encephalitis risk areas, Germany. Parasitology Research. 2011;108:323–35. 10.1007/s00436-010-2065-x 20878183 PMC3024494

[42] Brunner JL, Ostfeld RS. Multiple causes of variable tick burdens on small-mammal hosts. Ecology. 2008;89:2259–72. 18724736 10.1890/07-0665.1

[43] Krasnov BR, Morand S, Hawlena H, Khokhlova IS, Shenbrot GI. Sex-biased parasitism, seasonality and sexual size dimorphism in desert rodents. Oecologia. 2005;146:209–17. 10.1007/s00442-005-0189-y 16025350

[44] Kiffner C, Nagar S, Kollmar C, Kioko J. Wildlife species richness and densities in wildlife corridors of Northern Tanzania. Journal for Nature Conservation. 2016;31:29–37.

[45] Prins HHT, Loth PE. Rainfall patterns as background to plant phenology in northern Tanzania. Journal of Biogeography. 1988;15:451–463.

[46] Cohen AS, Halfpenny J, Lockley M, Michel E. Modern vertebrate tracks from Lake Manyara, Tanzania and their paleobiological implications. Paleobiology. 1993;19:433–58.

[47] Kiffner C, Kioko J, Kissui B, Painter C, Serota M, White C, et al Interspecific variation in large mammal responses to human observers along a conservation gradient with variable hunting pressure. Animal Conservation. 2014;17:603–12.

[48] Kiffner C, Peters L, Stroming A, Kioko J. Bushmeat consumption in the Tarangire-Manyara ecosystem, Tanzania. Tropical Conservation Science. 2015;8:318–32.

[49] Greenway JP, Vesey-Fitzgerald FD. The vegetation of Lake Manyara National Park. 1969;57:127–49.

[50] Myalyosi RBB. Influence of livestock grazing on range condition in south-west Masailand, northern Tanzania. Journal of Applied Ecology. 1992;29:581–8.

[51] Kiffner C, Rheault H, Miller E, Scheetz T, Enriquez V, Swafford R, et al Long-term population dynamics in a multi-species assemblage of large herbivores in East Africa. Ecosphere. 2017;8:e02027.

[52] Morrison TA, Link WA, Newmark WD, Foley CAH, Bolger DT. Tarangire revisited: Consequences of declining connectivity in a tropical ungulate population. Biological Conservation. 2016;197:53–60.

[53] Zimmerman DA, Turner DA, Pearson DJ. Birds of Kenya and Northern Tanzania. Princeton, New Jersey: Princeton University Press; 1999.

[54] Kiffner C, Kioko J, Leweri C, Krause S. Seasonal patterns of mixed species groups in large East African mammals. PLoS One 2014;9:e113446 10.1371/journal.pone.0113446 25470495 PMC4254287

[55] Warnes G, Ben Bolker, Lodewijk Bonebakker, Robert Gentleman, Wolfgang Huber, Andy Liaw, et al. gplots: Various R Programming Tools for Plotting Data. 3.0.1. ed2016.

[56] R Core Team. 2018 R: A language and environment for statistical computing. Vienna, Austria: R Foundation for Statistical Computing.

[57] Kingdon J. The Kingdon Field Guide to African Mammals. New York: Bloomsbury; 2012.

[58] Bourgarel M, Fritz H, Gaillard J-M, De Garine-Wichatitsky M, Maudet F. Effects of annual rainfall and habitat types on the body mass of Impala (*Aepyceros melampus*) in the Zambezi Valley Zimbabwe. African Journal of Ecology. 2002;40:186–93.

[59] Kashoma I, Luziga C, Werema CW, Shirima GA, D N. Predicting body weight of Tanzania shorthorn zebu cattle using heart girth measurement. Livestock Research for Rural Development 2011;23:Article 94.

[60] Mwakitwange G, Haule S, Massunga M. The status and potential of donkeys in the Southern Highlands of Tanzania In: Starkey P, Fielding D, editors. Donkeys, people and development. Amsterdam: Technical Centre for Agricultural and Rural Cooperation; 2004 p. 113–5.

[61] Wilson TR. Livestock production and farm animal genetic resources in the Usangu Wetland of the southern highlands of Tanzania. Livestock Research for Rural Development. 2003;15:Article 2.

[62] Wilson TR. Tanzania Southern Highlands Food Systems White Meat Value Chain Analysis. Food and Agriculture Organization of the United Nations; 2015.

[63] Buckland ST. Distance Sampling: Estimating Abundance of Biological Populations. 1st ed New York; London: Chapman & Hall; 1993.

[64] Grobler JH, Charsley GW. Host preference of the Yellow-billed Oxpecker *Buphagus africanus* in the Rhodes Matopos National Park, Rhodesia. South African Journal of Wildlife Research. 1978;8:169–70.

[65] Csardi G, Nepusz T. The igraph software package for complex network research. InterJournal, Complex Systems; 2006.

[66] Barton K. MuMIn: Multi-Model Inference. R package 1.40.0 ed2017.

[67] Richards SA. Dealing with overdispersed count data in applied ecology. Journal of Applied Ecology. 2008;45:218–27.

[68] Crawley MJ. The R Book. London: Imperial College London at Silwood Park; 2007.

[69] Keesing F, Young TP. Cascading consequences of the loss of large mammals in an African savanna. BioScience. 2014;64:487–95.

[70] Stutterheim IM, Bezuidenhout JD, Elliott EG. Comparative feeding behaviour and food preferences of oxpeckers (*Buphagus erythrorhynchus* and *B*. *africanus*) in captivity. Onderstepoort J Vet Res. 1988;55:173–9. 3194119

[71] Davidson Z, Valeix M, Loveridge AJ, Hunt JE, Johnson PJ, Madzikanda H, et al Environmental determinants of habitat and kill site selection in a large carnivore: scale matters. Journal of Mammalogy. 2012;93:677–85.

[72] Johnson DH. The comparison of usage and availability measurements for evaluating resource preference. Ecology. 1980;61:65–71.

[73] Lightfoot C, Norval RAI. Tick problems in wildlife in Zimbabwe. The effects of tick parasitism on wild ungulates. South African Journal of Wildlife Research 1981;11:41–5.

[74] Decker JE, McKay SD, Rolf MM, Kim J, Molina Alcalá A, Sonstegard TS, et al Worldwide patterns of ancestry, divergence, and admixture in domesticated cattle. PLOS Genetics. 2014;10: e1004254 10.1371/journal.pgen.1004254 24675901 PMC3967955

[75] Weeks P. Interactions between red-billed oxpeckers, *Buphagus erythrorhynchus*, and domestic cattle, *Bos taurus*, in Zimbabwe. Animal Behaviour. 1999;58:1253–9. 10.1006/anbe.1999.1265 10600147

[76] Keesing F, Allan BF, Young TP, Ostfeld RS. Effects of wildlife and cattle on tick abundance in central Kenya. Ecological Applications. 2013;23:1410–8. 24147412 10.1890/12-1607.1

[77] Mooring MS, Mundy PJ. Interactions between impala and oxpeckers at Matobo National Park, Zimbabwe. African Journal of Ecology. 1996;34:54–65.

[78] Grzeda E, Maurer T, Dannemann C, Kibiriti LO, Kioko J, Kiffner C. Effects of acaricide treatment and host intrinsic factors on tick acquisition and mortality in Boran cattle. Parasitology Research. 2017;116:3163–73. 10.1007/s00436-017-5633-5 28983669

[79] Warwick BT, Bak E, Baldassarre J, Gregg E, Martinez R, Kioko J, et al Abundance estimations of ixodid ticks on Boran cattle and Somali sheep in Northern Tanzania. International Journal of Acarology. 2016;42:12–7.

[80] Kioko J, Baker J, Shannon A, Kiffner C. Ethnoecological knowledge of ticks and treatment of tick-borne diseases among Maasai people in Northern Tanzania. Veterinary World. 2015;8:755–62. doi: 10.14202/vetworld.2015.755-762 27065643 10.14202/vetworld.2015.755-762 PMC4825278

[81] Tyrberg T. The Late Pleistocene continental avian extinction—an evaluation of the fossil evidence. Oryctos. 2008;7:249–69.

[82] Kalle R, Combrink L, Ramesh T, Downs CT. Re‐establishing the pecking order: Niche models reliably predict suitable habitats for the reintroduction of red‐billed oxpeckers. Ecology and Evolution. 2017;7:1974–83. 10.1002/ece3.2787 28331604 PMC5355191

[83] Ripple WJ, Chapron G, López-Bao JV, Durant SM, Macdonald DW, Lindsey PA, et al Conserving the world's megafauna and biodiversity: the fierce urgency of now. BioScience. 2017;67.

[84] Ripple WJ, Wolf C, Newsome TM, Hoffmann M, Wirsing AJ, McCauley DJ. Extinction risk is most acute for the world’s largest and smallest vertebrates. Proceedings of the National Academy of Sciences. 2017;114:10678–83. 10.1073/pnas.1702078114 PMC5635868 28923917

[85] Caughley G. Directions in conservation biology. Journal of Animal Ecology. 1994;63:215–44.

[86] Essl F, Dullinger S, Rabitsch W, Hulme PE, Pyšek P, Wilson JRU, et al Historical legacies accumulate to shape future biodiversity in an era of rapid global change. Diversity and Distributions. 2015;21:534–47.

