# Supplementary material for: Large mammal declines and the incipient loss of mammal-bird mutualisms in an African savanna ecosystem
Source: PLoS One. 2018 Aug 28;13(8):e0202536. doi: 10.1371/journal.pone.0202536 (PMC6112642; doi:10.1371/journal.pone.0202536)
Supplement: S1 Appendix — All oxpeckers observed on a host were recorded across 3 days of data collection in the Karatu District (KD), Burunge Wildlife Management Area (BWMA), Tarangire National Park (TNP), Mto wa Mbu Game Controlled Area (GCA), and Lake Manyara National Park (LMNP), and 4 days of data collection in Manyara Ranch (MR). (DOCX) [file pone.0202536.s001.docx]

| Species | Body Mass  (kg) | Total RBO Observed on Species | | | | | |  | Total YBO Total RBO Observed on Species | | | | | |
| --- | --- | --- | --- | --- | --- | --- | --- | --- | --- | --- | --- | --- | --- | --- |
|  |  | TNP | LMNP | BWMA | MR | GCA | KD |  | TNP | LMNP | BWMA | MR | GCA | KD |
| Bohor Reedbuck (*Redunca redunca*)^1^ | 47 | 0 | 0 | 0 | 1 | 0 | 0 |  | 0 | 0 | 0 | 0 | 0 | 0 |
| Buffalo (*Syncerus caffer*)^1^ | 555 | 6 | 180 | 0 | 11 | 0 | 0 |  | 11 | 1 | 0 | 0 | 0 | 0 |
| Bushbuck (*Tragelaphus sylvaticus*)^1^ | 48.5 | 0 | 2 | 0 | 0 | 0 | 0 |  | 0 | 0 | 0 | 0 | 0 | 0 |
| Cattle (*Bos spp.*)^2^ | 150 | 0 | 0 | 19 | 642 | 2333 | 4 |  | 0 | 0 | 8 | 0 | 0 | 0 |
| Dik-dik (*Madoqua kirkii*)^1^ | 5.5 | 0 | 5 | 0 | 21 | 0 | 0 |  | 0 | 0 | 0 | 0 | 0 | 0 |
| Donkey (*Equus africanus*)^3^ | 170 | 0 | 0 | 0 | 13 | 80 | 0 |  | 0 | 0 | 1 | 0 | 0 | 0 |
| Eland (*Taurotragus oryx*)^1^ | 560.5 | 0 | 0 | 0 | 57 | 0 | 0 |  | 0 | 0 | 0 | 0 | 0 | 0 |
| Elephant (*Loxodonta africana*)^1^ | 4000 | 0 | 85 | 0 | 31 | 0 | 0 |  | 0 | 0 | 0 | 0 | 0 | 0 |
| Giraffe (*Giraffa camelopardalis*)^1^ | 1340 | 132 | 26 | 55 | 123 | 0 | 0 |  | 22 | 0 | 11 | 13 | 0 | 0 |
| Grant’s gazelle (*Nanger granti*)^1^ | 61.5 | 0 | 1558 | 0 | 131 | 26 | 0 |  | 0 | 0 | 0 | 0 | 0 | 0 |
| Hippopotamus (*Hippopotamus amphibius*)^1^ | 1715 | 0 | 69 | 0 | 0 | 0 | 0 |  | 0 | 0 | 0 | 0 | 0 | 0 |
| Impala (*Aepyceros melampus*)^4^ | 56.25 | 42 | 596 | 5 | 363 | 13 | 0 |  | 0 | 0 | 0 | 0 | 0 | 0 |
| Klipspringer (*Oreotragus oreotragus*)^1^ | 13 | 0 | 1 | 0 | 0 | 0 | 0 |  | 0 | 0 | 0 | 0 | 0 | 0 |
| Lesser kudu (*Tragelaphus imberbis*)^1^ | 81.5 | 0 | 0 | 0 | 3 | 0 | 0 |  | 0 | 0 | 0 | 0 | 0 | 0 |
| Pig (*Sus domesticus*)^5^ | 56 | 0 | 0 | 0 | 0 | 0 | 0 |  | 0 | 0 | 0 | 0 | 0 | 0 |
| Sheep and Goat (*Capra spp.* and *Ovis spp.*)^6^ | 30 | 0 | 0 | 2 | 197 | 3744 | 0 |  | 0 | 0 | 0 | 0 | 0 | 0 |
| Steinbuck (*Raphicerus campestris*)^1^ | 11.5 | 0 | 0 | 0 | 1 | 0 | 0 |  | 0 | 0 | 0 | 0 | 0 | 0 |
| Thomson’s gazelle (*Eudorcas thomsoni*)^1^ | 23.75 | 0 | 0 | 0 | 210 | 279 | 0 |  | 0 | 0 | 0 | 0 | 0 | 0 |
| Warthog (*Phacochoerus africanus*)^1^ | 82.5 | 0 | 55 | 0 | 14 | 0 | 0 |  | 0 | 0 | 0 | 0 | 0 | 0 |
| Waterbuck (*Kobus e. ellipsiprymnus*)^1^ | 215 | 0 | 7 | 0 | 8 | 0 | 0 |  | 0 | 0 | 0 | 0 | 0 | 0 |
| Wildebeest (*Connochaetes taurinus*)^1^ | 213.75 | 0 | 407 | 0 | 88 | 190 | 0 |  | 0 | 0 | 0 | 0 | 0 | 0 |
| Zebra (*Equus quagga*)^1^ | 241.75 | 0 | 125 | 11 | 1744 | 0 | 0 |  | 0 | 0 | 1 | 15 | 0 | 0 |

*Body mass obtained from: ^1^[57], ^2^[59], ^3^[60], ^4^[58], ^5^[61], ^6^[62]
