# Supplementary material for: Large mammal declines and the incipient loss of mammal-bird mutualisms in an African savanna ecosystem
Source: PLoS One. 2018 Aug 28;13(8):e0202536. doi: 10.1371/journal.pone.0202536 (PMC6112642; doi:10.1371/journal.pone.0202536)
Supplement: S2 Appendix — (DOCX) [file pone.0202536.s002.docx]

| **Buffalo RBO** | | | | | | | | | |
| --- | --- | --- | --- | --- | --- | --- | --- | --- | --- |
| Intercept | Presence of Wound | Demographics | Time of Day | Group Size | df | logLik | AICc | Δ AICc | Weight |
| -1.7245 |  | + | + |  | 6 | -121.0783 | 254.3994 | 0.0000 | 0.4847 |
| -1.8514 |  | + | + | -0.3314 | 7 | -120.6892 | 255.7029 | 1.3036 | 0.2526 |
| -1.7427 | 0.1694 | + | + |  | 7 | -121.0690 | 256.4627 | 2.0633 | 0.1728 |
| -1.8634 | 0.1168 | + | + | -0.3296 | 8 | -120.6847 | 257.7881 | 3.3887 | 0.0891 |
| -0.3567 |  | + |  |  | 4 | -130.3666 | 268.8482 | 14.4488 | 0.0004 |
| -0.3670 |  | + |  | 0.3082 | 5 | -129.9124 | 269.9978 | 15.5984 | 0.0002 |
| -0.3688 | -0.8565 | + |  |  | 5 | -130.0507 | 270.2743 | 15.8749 | 0.0002 |
| -0.3768 | -0.7560 | + |  | 0.2831 | 6 | -129.6743 | 271.5913 | 17.1919 | 0.0001 |
| -3.4580 |  |  | + | -0.5741 | 4 | -139.8113 | 287.7376 | 33.3382 | 0.0000 |
| -3.4595 |  |  | + |  | 3 | -141.1981 | 288.4651 | 34.0657 | 0.0000 |
| -3.4858 | 1.0452 |  | + | -0.5474 | 5 | -139.5618 | 289.2964 | 34.8970 | 0.0000 |
| -3.4871 | 1.3388 |  | + |  | 4 | -140.8164 | 289.7478 | 35.3484 | 0.0000 |
| -1.3732 |  |  |  | -0.3951 | 2 | -177.3795 | 358.7933 | 104.3939 | 0.0000 |
| -1.3617 |  |  |  |  | 1 | -178.5656 | 359.1426 | 104.7432 | 0.0000 |
| -1.3736 | -0.3044 |  |  | -0.4067 | 3 | -177.3423 | 360.7533 | 106.3539 | 0.0000 |
| -1.3617 | -0.0250 |  |  |  | 2 | -178.5654 | 361.1650 | 106.7656 | 0.0000 |
| **Cattle RBO** | | | | | | | | | |
| Intercept | Presence of Wound | Demographics | Time of Day | Group Size | df | logLik | AICc | Δ AICc | Weight |
| -4.1067 |  | + | + | -0.7363 | 7 | -214.4368 | 442.8968 | 0.0000 | 0.3320 |
| -4.1290 | 1.2108 | + | + | -0.7446 | 8 | -213.4845 | 442.9989 | 0.1021 | 0.3155 |
| -4.0196 |  | + | + |  | 6 | -216.0673 | 444.1520 | 1.2553 | 0.1772 |
| -4.0405 | 1.1962 | + | + |  | 7 | -215.1380 | 444.2992 | 1.4024 | 0.1647 |
| -4.9628 |  | + |  | -0.7955 | 5 | -220.8323 | 451.6771 | 8.7803 | 0.0041 |
| -4.9915 | 1.1574 | + |  | -0.8034 | 6 | -219.9387 | 451.8948 | 8.9981 | 0.0037 |
| -4.9246 |  | + |  |  | 4 | -223.3317 | 454.6718 | 11.7750 | 0.0009 |
| -4.9453 | 1.1196 | + |  |  | 5 | -222.4885 | 454.9894 | 12.0926 | 0.0008 |
| -4.0100 | 1.2637 |  | + |  | 4 | -224.4835 | 456.9753 | 14.0785 | 0.0003 |
| -4.0023 |  |  | + |  | 3 | -225.5304 | 457.0657 | 14.1689 | 0.0003 |
| -4.1107 |  |  | + | -0.5254 | 4 | -224.6964 | 457.4011 | 14.5043 | 0.0002 |
| -4.1132 | 1.2308 |  | + | -0.5120 | 5 | -223.6960 | 457.4045 | 14.5077 | 0.0002 |
| -4.8354 |  |  |  | -0.6895 | 2 | -229.6075 | 463.2174 | 20.3206 | 0.0000 |
| -4.8497 | 1.1099 |  |  | -0.6797 | 3 | -228.7637 | 463.5323 | 20.6355 | 0.0000 |
| -4.7829 |  |  |  |  | 1 | -231.4829 | 464.9666 | 22.0698 | 0.0000 |
| -4.7986 | 1.1542 |  |  |  | 2 | -230.5796 | 465.1616 | 22.2648 | 0.0000 |
| **Eland RBO** | | | | | | | | | |
| Intercept | Presence of Wound | Demographics | Time of Day | Group Size | df | logLik | AICc | Δ AICc | Weight |
| -2.0706 |  | + | + | -2.2834 | 6 | -21.7453 | 57.1705 | 0.0000 | 0.3334 |
| -1.4327 |  | + | + |  | 5 | -23.7449 | 58.6663 | 1.4958 | 0.1578 |
| -1.1150 | 20.1758 | + | + |  | 6 | -22.7438 | 59.1676 | 1.9972 | 0.1228 |
| -2.0889 |  |  | + | -3.5565 | 4 | -25.2796 | 59.3284 | 2.1580 | 0.1133 |
| -1.6753 | 17.5527 | + | + | -1.9699 | 7 | -21.6452 | 59.5761 | 2.4056 | 0.1001 |
| -0.8693 | 19.7825 | + |  |  | 4 | -26.0244 | 60.8179 | 3.6474 | 0.0538 |
| -1.0986 |  | + |  |  | 3 | -27.4544 | 61.3616 | 4.1911 | 0.0410 |
| -1.7942 | 14.6850 |  | + | -3.4648 | 5 | -25.2605 | 61.6975 | 4.5270 | 0.0347 |
| -0.9266 | 20.2696 | + |  | 0.4884 | 5 | -25.8172 | 62.8109 | 5.6404 | 0.0199 |
| -1.1040 |  | + |  | 0.0630 | 4 | -27.4503 | 63.6698 | 6.4993 | 0.0129 |
| -0.6931 |  |  | + |  | 3 | -29.7064 | 65.8657 | 8.6952 | 0.0043 |
| -0.3553 | 19.2592 |  | + |  | 4 | -28.6431 | 66.0555 | 8.8850 | 0.0039 |
| -0.7148 | 16.5716 |  |  |  | 2 | -32.5425 | 69.3072 | 12.1367 | 0.0008 |
| -0.9410 |  |  |  |  | 1 | -33.8361 | 69.7448 | 12.5743 | 0.0006 |
| -0.9627 |  |  |  | -0.6387 | 2 | -33.2658 | 70.7539 | 13.5834 | 0.0004 |
| -0.7256 | 16.1893 |  |  | -0.4478 | 3 | -32.2826 | 71.0180 | 13.8475 | 0.0003 |
| **Giraffe RBO** | | | | | | | | | |
| Intercept | Presence of Wound | Demographics | Time of Day | Group Size | df | logLik | AICc | Δ AICc | Weight |
| -0.4502 | 0.8740 | + |  | -0.8243 | 6 | -168.6091 | 349.5182 | 0.0000 | 0.3244 |
| -0.4572 |  | + |  | -0.9112 | 5 | -170.0470 | 350.3076 | 0.7894 | 0.2186 |
| -0.2753 | 0.9910 | + |  |  | 5 | -170.4318 | 351.0771 | 1.5588 | 0.1488 |
| -0.6464 | 0.9367 |  |  | -1.5728 | 3 | -173.0981 | 352.2810 | 2.7627 | 0.0815 |
| -0.2624 |  | + |  |  | 4 | -172.3243 | 352.7904 | 3.2722 | 0.0632 |
| -0.6516 |  |  |  | -1.6635 | 2 | -174.7873 | 353.6169 | 4.0987 | 0.0418 |
| -0.4095 | 0.8619 | + | + | -0.8019 | 8 | -168.5604 | 353.6389 | 4.1206 | 0.0413 |
| -0.4099 |  | + | + | -0.8784 | 7 | -169.9497 | 354.3008 | 4.7826 | 0.0297 |
| -0.2019 | 0.9606 | + | + |  | 7 | -170.2248 | 354.8511 | 5.3328 | 0.0225 |
| -0.1802 |  | + | + |  | 6 | -171.9822 | 356.2643 | 6.7461 | 0.0111 |
| -0.5639 | 0.9313 |  | + | -1.5541 | 5 | -173.0292 | 356.2719 | 6.7537 | 0.0111 |
| -0.5585 |  |  | + | -1.6367 | 4 | -174.6900 | 357.5218 | 8.0036 | 0.0059 |
| -0.5390 | 1.3146 |  |  |  | 2 | -185.5274 | 375.0970 | 25.5787 | 0.0000 |
| -0.2650 | 1.2380 |  | + |  | 4 | -184.0717 | 376.2853 | 26.7671 | 0.0000 |
| -0.5351 |  |  |  |  | 1 | -189.0189 | 380.0518 | 30.5336 | 0.0000 |
| -0.2364 |  |  | + |  | 3 | -187.1073 | 380.2994 | 30.7812 | 0.0000 |
| **Impala RBO** | | | | | | | | | |
| Intercept | Presence of Wound | Demographics | Time of Day | Group Size | df | logLik | AICc | Δ AICc | Weight |
| -3.3882 |  |  |  |  | 1 | -273.1043 | 548.2107 | 0.0000 | 0.2350 |
| -3.3985 |  |  |  | 0.2947 | 2 | -272.4280 | 548.8623 | 0.6516 | 0.1696 |
| -3.4293 | -12.1817 |  |  |  | 2 | -272.8714 | 549.7491 | 1.5384 | 0.1089 |
| -3.1676 |  |  | + |  | 3 | -272.0031 | 550.0188 | 1.8082 | 0.0951 |
| -3.4396 | -12.1449 |  |  | 0.2935 | 3 | -272.2024 | 550.4175 | 2.2068 | 0.0779 |
| -3.2009 |  | + |  |  | 4 | -271.3709 | 550.7630 | 2.5524 | 0.0656 |
| -3.2047 |  |  | + | 0.2479 | 4 | -271.5469 | 551.1150 | 2.9043 | 0.0550 |
| -3.2090 | -12.1827 |  | + |  | 4 | -271.7691 | 551.5593 | 3.3486 | 0.0440 |
| -3.2372 |  | + |  | 0.1958 | 5 | -271.1106 | 552.2529 | 4.0422 | 0.0311 |
| -3.2517 | -14.0570 | + |  |  | 5 | -271.1647 | 552.3611 | 4.1504 | 0.0295 |
| -3.2457 | -12.1497 |  | + | 0.2464 | 5 | -271.3199 | 552.6715 | 4.4609 | 0.0253 |
| -3.0203 |  | + | + |  | 6 | -270.3692 | 552.7828 | 4.5721 | 0.0239 |
| -3.2878 | -14.0442 | + |  | 0.1951 | 6 | -270.9069 | 553.8583 | 5.6476 | 0.0140 |
| -3.0710 | -14.0725 | + | + |  | 7 | -270.1589 | 554.3770 | 6.1663 | 0.0108 |
| -3.0596 |  | + | + | 0.1407 | 7 | -270.2413 | 554.5419 | 6.3312 | 0.0099 |
| -3.1100 | -14.0605 | + | + | 0.1394 | 8 | -270.0337 | 556.1437 | 7.9330 | 0.0044 |
| **Wildebeest RBO** | | | | | | | | | |
| Intercept | Presence of Wound | Demographics | Time of Day | Group Size | df | logLik | AICc | Δ AICc | Weight |
| -17.6407 |  |  |  | -23.1293 | 2 | -10.2226 | 24.4581 | 0.0000 | 0.4531 |
| -17.6232 | 16.4823 |  |  | -23.1293 | 3 | -10.2226 | 26.4710 | 2.0128 | 0.1656 |
| -34.6403 |  |  | + | -24.0782 | 4 | -9.5626 | 27.1680 | 2.7099 | 0.1169 |
| -23.5661 |  | + |  |  | 4 | -10.1551 | 28.3529 | 3.8948 | 0.0646 |
| -27.3908 |  | + |  | -8.5029 | 5 | -9.4655 | 28.9953 | 4.5372 | 0.0469 |
| -34.6264 | 12.4171 |  | + | -24.0782 | 5 | -9.5626 | 29.1895 | 4.7314 | 0.0425 |
| -23.5661 | 0.0000 | + |  |  | 5 | -10.1551 | 30.3744 | 5.9163 | 0.0235 |
| -6.1495 |  |  |  |  | 1 | -14.3012 | 30.6067 | 6.1486 | 0.0209 |
| -27.3846 | 8.8599 | + |  | -8.5029 | 6 | -9.4655 | 31.0212 | 6.5630 | 0.0170 |
| -43.0532 |  | + | + |  | 6 | -9.6276 | 31.3454 | 6.8873 | 0.0145 |
| -46.5358 |  | + | + | -9.4085 | 7 | -8.8319 | 31.7842 | 7.3261 | 0.0116 |
| -6.1596 | -10.4176 |  |  |  | 2 | -14.2991 | 32.6110 | 8.1528 | 0.0077 |
| -43.0530 | -0.0872 | + | + |  | 7 | -9.6276 | 33.3756 | 8.9174 | 0.0052 |
| -46.5283 | 9.8859 | + | + | -9.4085 | 8 | -8.8319 | 33.8187 | 9.3606 | 0.0042 |
| -21.5661 |  |  | + |  | 3 | -13.9069 | 33.8394 | 9.3813 | 0.0042 |
| -21.5825 | -15.4197 |  | + |  | 4 | -13.9047 | 35.8523 | 11.3942 | 0.0015 |
| **Zebra RBO** | | | | | | | | | |
| Intercept | Presence of Wound | Demographics | Time of Day | Group Size | df | logLik | AICc | Δ AICc | Weight |
| -4.0347 |  |  |  |  | 1 | -181.5669 | 365.1358 | 0.0000 | 0.3364 |
| -4.0377 |  |  |  | -0.1614 | 2 | -181.4612 | 366.9283 | 1.7925 | 0.1373 |
| -4.0362 | 0.3800 |  |  |  | 2 | -181.5057 | 367.0172 | 1.8814 | 0.1313 |
| -3.7913 |  |  | + |  | 3 | -180.7038 | 367.4191 | 2.2833 | 0.1074 |
| -4.0390 | 0.3572 |  |  | -0.1563 | 3 | -181.4069 | 368.8253 | 3.6896 | 0.0532 |
| -3.8558 |  | + |  |  | 4 | -180.4615 | 368.9424 | 3.8066 | 0.0502 |
| -3.7955 |  |  | + | -0.2135 | 4 | -180.5252 | 369.0699 | 3.9341 | 0.0471 |
| -3.7944 | 0.3631 |  | + |  | 4 | -180.6477 | 369.3147 | 4.1789 | 0.0416 |
| -3.8672 |  | + |  | -0.1387 | 5 | -180.3864 | 370.8018 | 5.6660 | 0.0198 |
| -3.8573 | 0.3108 | + |  |  | 5 | -180.4202 | 370.8694 | 5.7337 | 0.0191 |
| -3.7984 | 0.3362 |  | + | -0.2091 | 5 | -180.4769 | 370.9828 | 5.8471 | 0.0181 |
| -3.6099 |  | + | + |  | 6 | -179.6027 | 371.2461 | 6.1103 | 0.0158 |
| -3.8683 | 0.2960 | + |  | -0.1354 | 6 | -180.3488 | 372.7383 | 7.6025 | 0.0075 |
| -3.6213 |  | + | + | -0.1925 | 7 | -179.4617 | 372.9778 | 7.8420 | 0.0067 |
| -3.6125 | 0.2948 | + | + |  | 7 | -179.5655 | 373.1852 | 8.0494 | 0.0060 |
| -3.6236 | 0.2776 | + | + | -0.1899 | 8 | -179.4286 | 374.9270 | 9.7912 | 0.0025 |
| **Buffalo YBO** | | | | | | | | | |
| Intercept | Presence of Wound | Demographics | Time of Day | Group Size | df | logLik | AICc | Δ AICc | Weight |
| -0.5190 | -21.9119 | + | + | 3.1307 | 8 | -16.4930 | 49.4045 | 0.0000 | 0.5983 |
| -0.3715 |  | + | + | 2.6766 | 7 | -17.9704 | 50.2654 | 0.8608 | 0.3890 |
| -0.6365 |  | + | + |  | 6 | -23.0537 | 58.3501 | 8.9456 | 0.0068 |
| -0.8726 | -19.8128 | + | + |  | 7 | -22.2092 | 58.7430 | 9.3385 | 0.0056 |
| -3.4032 |  | + |  | 2.7248 | 5 | -27.7525 | 65.6778 | 16.2733 | 0.0002 |
| -3.6829 | -18.2829 | + |  | 2.8172 | 6 | -27.5086 | 67.2599 | 17.8554 | 0.0001 |
| -3.0082 |  | + |  |  | 4 | -31.0274 | 70.1697 | 20.7652 | 0.0000 |
| -3.2722 | -18.6441 | + |  |  | 5 | -30.7560 | 71.6849 | 22.2804 | 0.0000 |
| -3.7641 |  |  |  |  | 1 | -38.2049 | 78.4211 | 29.0166 | 0.0000 |
| -3.8975 |  |  |  | 1.1622 | 2 | -37.3395 | 78.7133 | 29.3088 | 0.0000 |
| -3.2708 |  |  | + |  | 3 | -36.7260 | 79.5208 | 30.1162 | 0.0000 |
| -3.9452 | -13.8166 |  |  |  | 2 | -38.0894 | 80.2131 | 30.8086 | 0.0000 |
| -4.0712 | -13.1584 |  |  | 1.1403 | 3 | -37.2752 | 80.6192 | 31.2146 | 0.0000 |
| -3.4029 |  |  | + | 0.7546 | 4 | -36.3069 | 80.7288 | 31.3243 | 0.0000 |
| -3.4614 | -14.8050 |  | + |  | 4 | -36.6019 | 81.3188 | 31.9142 | 0.0000 |
| -3.5869 | -14.3777 |  | + | 0.7264 | 5 | -36.2244 | 82.6218 | 33.2172 | 0.0000 |
| **Cattle YBO** | | | | | | | | | |
| Intercept | Presence of Wound | Demographics | Time of Day | Group Size | df | logLik | AICc | Δ AICc | Weight |
| -25.1906 |  | + | + | 1.9173 | 7 | -34.4318 | 82.8868 | 0.0000 | 0.3581 |
| -8.6636 |  | + |  | 2.8229 | 5 | -36.7463 | 83.5051 | 0.6183 | 0.2629 |
| -25.5199 | -18.9014 | + | + | 1.9555 | 8 | -33.9816 | 83.9931 | 1.1063 | 0.2060 |
| -8.9985 | -17.8151 | + |  | 2.8858 | 6 | -36.3249 | 84.6673 | 1.7805 | 0.1470 |
| -24.8321 |  | + | + |  | 6 | -38.4806 | 88.9786 | 6.0918 | 0.0170 |
| -25.1405 | -18.7271 | + | + |  | 7 | -38.1369 | 90.2971 | 7.4103 | 0.0088 |
| -7.3291 |  | + |  |  | 4 | -45.0760 | 98.1603 | 15.2734 | 0.0002 |
| -7.5959 | -17.2656 | + |  |  | 5 | -44.8827 | 99.7779 | 16.8911 | 0.0001 |
| -7.5344 |  |  |  | 2.7665 | 2 | -50.8294 | 105.6612 | 22.7744 | 0.0000 |
| -23.3224 |  |  | + | 1.9142 | 4 | -48.9095 | 105.8272 | 22.9404 | 0.0000 |
| -7.7513 | -13.8010 |  |  | 2.7696 | 3 | -50.7121 | 107.4292 | 24.5424 | 0.0000 |
| -23.5848 | -16.8670 |  | + | 1.9085 | 5 | -48.7836 | 107.5797 | 24.6929 | 0.0000 |
| -23.5661 |  |  | + |  | 3 | -52.8721 | 111.7492 | 28.8624 | 0.0000 |
| -23.8376 | -17.3001 |  | + |  | 4 | -52.6980 | 113.4043 | 30.5175 | 0.0000 |
| -6.3990 |  |  |  |  | 1 | -59.1987 | 120.3983 | 37.5115 | 0.0000 |
| -6.6011 | -13.1838 |  |  |  | 2 | -59.0647 | 122.1318 | 39.2450 | 0.0000 |
| **Giraffe YBO** | | | | | | | | | |
| Intercept | Presence of Wound | Demographics | Time of Day | Group Size | df | logLik | AICc | Δ AICc | Weight |
| -6.5848 |  |  | + |  | 3 | -63.4135 | 132.8386 | 0.0000 | 0.3407 |
| -6.6584 |  |  | + | 0.8745 | 4 | -62.7862 | 133.5917 | 0.7531 | 0.2338 |
| -6.8373 | -14.3038 |  | + |  | 4 | -63.1858 | 134.3910 | 1.5524 | 0.1568 |
| -6.9082 | -14.1751 |  | + | 0.8565 | 5 | -62.5832 | 135.1954 | 2.3567 | 0.1049 |
| -7.4040 |  | + | + |  | 6 | -62.2065 | 136.4537 | 3.6151 | 0.0559 |
| -7.4200 |  | + | + | 0.8134 | 7 | -61.6695 | 137.3933 | 4.5547 | 0.0349 |
| -7.6525 | -14.2823 | + | + |  | 7 | -61.9802 | 138.0147 | 5.1761 | 0.0256 |
| -7.6681 | -14.1784 | + | + | 0.7970 | 8 | -61.4626 | 138.9950 | 6.1564 | 0.0157 |
| -5.2326 |  |  |  |  | 1 | -68.5875 | 139.1770 | 6.3384 | 0.0143 |
| -5.4709 | -13.3531 |  |  |  | 2 | -68.3724 | 140.7507 | 7.9120 | 0.0065 |
| -5.2333 |  |  |  | 0.0748 | 2 | -68.5796 | 141.1650 | 8.3264 | 0.0053 |
| -5.4714 | -13.3429 |  |  | 0.0627 | 3 | -68.3668 | 142.7453 | 9.9066 | 0.0024 |
| -5.8889 |  | + |  |  | 4 | -67.7836 | 143.5865 | 10.7479 | 0.0016 |
| -6.1264 | -13.3855 | + |  |  | 5 | -67.5579 | 145.1448 | 12.3062 | 0.0007 |
| -5.8866 |  | + |  | 0.0334 | 5 | -67.7820 | 145.5931 | 12.7545 | 0.0006 |
| -6.1248 | -13.3824 | + |  | 0.0223 | 6 | -67.5572 | 147.1550 | 14.3164 | 0.0003 |
| **Zebra YBO** | | | | | | | | | |
| Intercept | Presence of Wound | Demographics | Time of Day | Group Size | df | logLik | AICc | Δ AICc | Weight |
| -4.0347 |  |  |  |  | 1 | -181.5669 | 365.1358 | 0.0000 | 0.3364 |
| -4.0377 |  |  |  | -0.1614 | 2 | -181.4612 | 366.9283 | 1.7925 | 0.1373 |
| -4.0362 | 0.3800 |  |  |  | 2 | -181.5057 | 367.0172 | 1.8814 | 0.1313 |
| -3.7913 |  |  | + |  | 3 | -180.7038 | 367.4191 | 2.2833 | 0.1074 |
| -4.0390 | 0.3572 |  |  | -0.1563 | 3 | -181.4069 | 368.8253 | 3.6896 | 0.0532 |
| -3.8558 |  | + |  |  | 4 | -180.4615 | 368.9424 | 3.8066 | 0.0502 |
| -3.7955 |  |  | + | -0.2135 | 4 | -180.5252 | 369.0699 | 3.9341 | 0.0471 |
| -3.7944 | 0.3631 |  | + |  | 4 | -180.6477 | 369.3147 | 4.1789 | 0.0416 |
| -3.8672 |  | + |  | -0.1387 | 5 | -180.3864 | 370.8018 | 5.6660 | 0.0198 |
| -3.8573 | 0.3108 | + |  |  | 5 | -180.4202 | 370.8694 | 5.7337 | 0.0191 |
| -3.7984 | 0.3362 |  | + | -0.2091 | 5 | -180.4769 | 370.9828 | 5.8471 | 0.0181 |
| -3.6099 |  | + | + |  | 6 | -179.6027 | 371.2461 | 6.1103 | 0.0158 |
| -3.8683 | 0.2960 | + |  | -0.1354 | 6 | -180.3488 | 372.7383 | 7.6025 | 0.0075 |
| -3.6213 |  | + | + | -0.1925 | 7 | -179.4617 | 372.9778 | 7.8420 | 0.0067 |
| -3.6125 | 0.2948 | + | + |  | 7 | -179.5655 | 373.1852 | 8.0494 | 0.0060 |
| -3.6236 | 0.2776 | + | + | -0.1899 | 8 | -179.4286 | 374.9270 | 9.7912 | 0.0025 |
