# Supplementary material for: Large mammal declines and the incipient loss of mammal-bird mutualisms in an African savanna ecosystem
Source: PLoS One. 2018 Aug 28;13(8):e0202536. doi: 10.1371/journal.pone.0202536 (PMC6112642; doi:10.1371/journal.pone.0202536)
Supplement: S3 Appendix — All host individuals were recorded across 3 days of data collection in the Karatu District (KD), Burunge Wildlife Management Area (BWMA), Tarangire National Park (TNP), Mto wa Mbu Game Controlled Area (GCA), and 4 days of data collection in Manyara Ranch (MR). (DOCX) [file pone.0202536.s003.docx]

| Species | Body Mass  (kg) | Total Individuals Observed | | | | | | Percent Relative Host Abundance | | | | | |
| --- | --- | --- | --- | --- | --- | --- | --- | --- | --- | --- | --- | --- | --- |
|  |  | TNP | LMNP | BWMA | MR | GCA | KD | TNP | LMNP | BWMA | MR | GCA | KD |
| Bohor Reedbuck (*Redunca redunca*)^1^ | 47 | 0 | 0 | 0 | 1 | 0 | 0 | 0 | 0 | 0 | 0 | 0 | 0 |
| Buffalo (*Syncerus caffer*)^1^ | 555 | 162 | 180 | 0 | 11 | 0 | 0 | 13.0 | 11.6 | 0 | 0.3 | 0 | 0 |
| Bushbuck (*Tragelaphus sylvaticus*)^1^ | 48.5 | 0 | 2 | 0 | 0 | 0 | 0 | 0 | 0.1 | 0 | 0 | 0 | 0 |
| Cattle (*Bos spp.*)^2^ | 150 | 0 | 0 | 1014 | 642 | 2333 | 8290 | 0 | 0 | 36.1 | 17.6 | 35.0 | 43.4 |
| Dik-dik (*Madoqua kirkii*)^1^ | 5.5 | 9 | 5 | 22 | 21 | 0 |  | 0.7 | 0.3 | 0.8 | 0.6 | 0 | 0 |
| Donkey (*Equus africanus*)^3^ | 170 | 0 | 0 | 31 | 13 | 80 | 66 | 0 | 0 | 1.1 | 0.4 | 1.2 | 3.5 |
| Eland (*Taurotragus oryx*)^1^ | 560.5 | 0 | 0 | 0 | 57 | 0 | 0 | 0 | 0 | 0 | 1.6 | 0 | 0 |
| Elephant (*Loxodonta africana*)^1^ | 4000 | 189 | 85 | 58 | 31 | 0 | 0 | 15.1 | 5.5 | 2.1 | 0.8 | 0 | 0 |
| Giraffe (*Giraffa camelopardalis*)^1^ | 1340 | 66 | 26 | 72 | 123 | 0 | 0 | 5.3 | 1.7 | 2.6 | 3.4 | 0 | 0 |
| Grant’s gazelle (*Nanger granti*)^1^ | 61.5 | 12 | 1558 | 0 | 131 | 26 | 0 | 1.0 | 0 | 0 | 3.6 | 0.4 | 0 |
| Hippopotamus (*Hippopotamus amphibius*)^1^ | 1715 | 0 | 69 | 0 | 0 | 0 | 0 | 0 | 4.4 | 0 | 0 | 0 | 0 |
| Impala (*Aepyceros melampus*)^4^ | 56.25 | 729 | 596 | 197 | 363 | 13 | 0 | 58.4 | 38.3 | 7.0 | 9.9 | 0.2 | 0 |
| Klipspringer (*Oreotragus oreotragus*)^1^ | 13 | 0 | 1 | 0 | 0 | 0 | 0 | 0 | 0.1 | 0 | 0 | 0 | 0 |
| Lesser kudu (*Tragelaphus imberbis*)^1^ | 81.5 | 0 | 0 | 4 | 3 | 0 | 0 | 0 | 0 | 0.1 | 0.1 | 0 | 0 |
| Pig (*Sus domesticus*)^5^ | 56 | 0 | 0 | 0 | 0 |  | 10 | 0 | 0 | 0 | 0 | 0 | 0.5 |
| Sheep and Goat (*Capra spp.* and *Ovis spp.*)^6^ | 30 | 0 | 0 | 924 | 197 | 3744 | 1007 | 0 | 0 | 32.9 | 5.4 | 56.2 | 52.7 |
| Steinbuck (*Raphicerus campestris*)^1^ | 11.5 | 0 | 0 | 0 | 1 | 0 | 0 | 0 | 0 | 0 | 0 | 0 | 0 |
| Thomson’s gazelle (*Eudorcas thomsoni*)^1^ | 23.75 | 0 | 0 | 0 | 210 | 279 | 0 | 0 | 0 | 0 | 5.7 | 4.2 | 0 |
| Warthog (*Phacochoerus africanus*)^1^ | 82.5 | 47 | 55 | 29 | 14 | 0 | 0 | 3.8 | 3.5 | 1.0 | 0.4 | 0 | 0 |
| Waterbuck (*Kobus e. ellipsiprymnus*)^1^ | 215 | 34 | 7 | 5 | 8 | 0 | 0 | 2.7 | 0.4 | 0.2 | 0.2 | 0 | 0 |
| Wildebeest (*Connochaetes taurinus*)^1^ | 213.75 | 0 | 407 | 254 | 88 | 190 | 0 | 0 | 26.1 | 9.0 | 2.4 | 2.9 | 0 |
| Zebra (*Equus quagga*)^1^ | 241.75 | 0 | 125 | 202 | 1744 | 0 | 0 | 0 | 8.0 | 7.2 | 47.7 | 0 | 0 |

*Body mass obtained from: ^1^[57], ^2^[59], ^3^[60], ^4^[58], ^5^[61], ^6^[62]
